# Structural determinants of DNA cleavage by a CRISPR HNH-Cascade system

**DOI:** 10.1016/j.molcel.2024.07.026

**Published:** 2024-08-06

**Authors:** Seiichi Hirano, Han Altae-Tran, Soumya Kannan, Rhiannon K. Macrae, Feng Zhang

**Affiliations:** 1 Broad Institute of MIT and Harvard, Cambridge, MA 02142, US; 2 McGovern Institute for Brain Research at MIT, Cambridge, MA 02139, USA; 3 Department of Brain and Cognitive Science, Massachusetts Institute of Technology, Cambridge, MA 02139, USA; 4 Department of Biological Engineering, Massachusetts Institute of Technology, Cambridge, MA 02139, USA; 5 Howard Hughes Medical Institute, Cambridge, MA 02139, USA; 6 Lead Contact

**Keywords:** CRISPR, cryo-EM, HNH, DNA endonuclease, Ribonuclease, Cascade, Bacterial immune system, DNA nicking, Programmable nuclease, Molecular adaptation, Macromolecular complex

## Abstract

Canonical prokaryotic type I CRISPR-Cas adaptive immune systems contain a multicomponent effector complex called Cascade, which degrades large stretches of DNA via Cas3 helicase-nuclease activity. Recently, a highly precise subtype I-F1 CRISPR-Cas system (HNH-Cascade) was found that lacks Cas3, the absence of which is compensated for by the insertion of an HNH endonuclease domain in the Cas8 Cascade component. Here, we describe the cryo-EM structure of *Selenomonas* sp. HNH-Cascade (SsCascade) in complex with target DNA and characterize its mechanism of action. The Cascade scaffold is complemented by the HNH domain, creating a ring-like structure in which the unwound target DNA is precisely cleaved. This structure visualizes a unique hybrid of two extensible biological systems — Cascade, an evolutionary platform for programmable DNA effectors, and an HNH nuclease, an adaptive domain with a spectrum of enzymatic activity.

## Introduction

CRISPR-Cas systems in prokaryotes are a diverse group of systems that mediate RNA-guided adaptive immunity, targeting viral and other foreign nucleic acids with variable biological consequences, such as nucleic acid cleavage and degradation^1^. The most prevalent of these systems (~60%) are the type I CRISPR-Cas systems, which utilize multiple Cas proteins that are assembled into a crRNA-containing complex known as Cascade^2^. In canonical type I systems, the endonuclease-helicase Cas3 is the catalytic module in Cascade, mediating DNA degradation^3^. A recent study reported a new variant of type I systems, related to the I-F subtype, that consists of Cas5f1/6f/7f1/8f1 (formerly Csy2/4/3/1, referred to as Cas5/6/7/8 for simplicity) and a CRISPR RNA (crRNA), but which lacks Cas3^4^. Additionally, there is an HNH endonuclease domain inserted into the C-terminus of Cas8. Previous work has shown that this system, referred to as HNH-Cascade, cleaves double- and single-stranded DNA in a Cas8-HNH-dependent manner^4^.

HNH-mediated cleavage of DNA is also seen with Cas9, the single effector module of type II CRISPR-Cas systems. Cas9 cleaves double-stranded DNA, utilizing the HNH domain to cleave the target strand and a RuvC endonuclease domain to cleave the non-target strand^5,6^. However, the mechanism of DNA targeting and cleavage of HNH-Cascade is unknown. To elucidate its mechanism of action, we determined the cryo-EM structure of *Selenomonas* sp. HNH-Cascade (SsCascade) in complex with target DNA, revealing a ring-like ribonucleoprotein architecture. We also performed biochemical experiments to dissect the activity of this unusual system.

## Results

### Overall structure of Cascade

**Figure 1.**
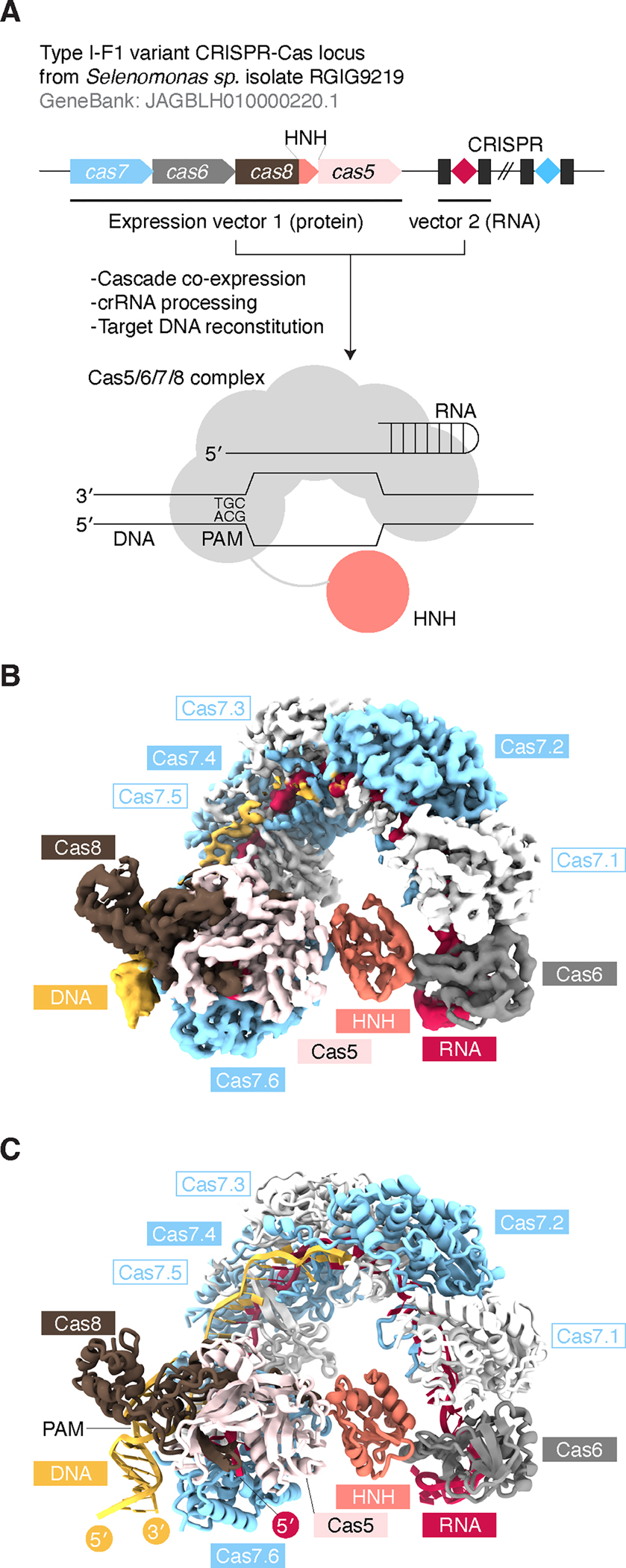
Cryo-EM structure of the SsCascade-target DNA complex (A) Locus architecture and reconstitution of *Selenomonas* sp. HNH-Cascade (SsCascade). (B and C) Cryo-EM-density map (B) and structural model (C) of the SsCascade-target DNA complex. See also Figures S1–S6, Movie S1.

We determined the cryo-EM structure of target DNA-bound SsCascade containing Cas5/6/7/8 proteins and a processed crRNA at 3.48Å resolution (Map A) (Figure 1, Figures S1–3, and Table 1). SsCascade forms a “seahorse”-shaped architecture containing head-trunk-tail parts, which are common in typical Cascade structures^7,8^ (Figure 1C and Figure S3A). The head part is capped by Cas6 binding to the crRNA 3′-hairpin region. The trunk runs along the crRNA spacer region supported by six Cas7 molecules (Cas7.1–7.6). The tail part consists of a Cas5-Cas8 heterodimer which accommodates the crRNA 5′-handle region and the PAM-containing DNA duplex. Starting from the PAM-proximal region, an 18-nt target strand DNA base-pairing with crRNA and a 2-nt non-target strand DNA are observed in the density map, while the remaining DNA part of a putative R-loop and the cleavage sites-containing DNA duplex are disordered. Further single-particle analysis enabled us to observe the crRNA 3′-hairpin–Cas6 displacement in the sub-clustered density maps, indicating flexibility in the head region (Figure S1C and Movie S1). By contrast, the merging process of different particle subsets improved the density map quality of the trunk and tail parts up to 3.28Å resolution (Map B), indicating these regions are rigid and may play a scaffolding role for DNA targeting. The C-terminal HNH domain of Cas8 is located between the head and tail parts and is clearly observed in Map A, the head-focused refinement map, but not in Map B, the overall refinement map (Figure S1C). HNH and Cas6 show continuous EM density in the sub-clustered density maps, indicating that the HNH domain is associated with the head movement (Movie S1). Taken together, these structures reveal that HNH-Cascade adopts the typical DNA-targeting scaffold of other Cascade complexes, with the addition of a flexibly positioned HNH domain.

### crRNA-mediated Cascade assembly

The Cascade crRNA is transcribed from the CRISPR array and processed by an endoribonuclease, Cas6, which triggers the ribonucleoprotein complex formation in type I-E and F Cascades^9–11^. Consistent with this, our structure shows that Cas6 binds to the crRNA 3′-hairpin through the interactions between the positively charged ɑ-helix and the major groove of the RNA helix (Figure S4A). Cas6 consists of an N-terminal ferredoxin-like domain and a C-terminal domain which is adapted into RNA hairpin binding (Figure S3C). These two domains accommodate the crRNA 3′-end, and the conserved residues (His29 and Ser148) in these domains are responsible for the crRNA processing^10^ (Figure S4).

All Cas7 subunits (Cas7.1–7.6) adopt a conserved “right-hand” architecture consisting of the fingers-, palm-, web-, and thumb-shaped domains^7,8^ (Figure S3E). The Cas7 interaction manner with other molecules defines the structural divergence of Cascade. The Cas7.1 finger domain has minimal contacts with the two loops of the Cas6 N-terminal domain in our structure of SsCascade, while the additional contacts between the Cas7.1 thumb and the Cas6 C-terminal domain change the Cas6 orientation in Cascade from *Pseudomonas aeruginosa*^12,13^ (PaCascade, another Cascade system coupled with Cas3) (Figure S5A). The palm/web/thumb domains of the Cas7.1–7.6 subunits distort periodically kinked spacer parts of the crRNA (Figure S5B). The torsion angle at each kink results in the tighter helical pitch of SsCascade compared to those of PaCascade and another Cascade from *Vibrio cholerae*^12,14^ (VcCascade, a transposase-associated Cascade), consistent with head-to-tail communication within SsCascade (which has no additional components) vs Pa/VcCascade (which additionally contain Cas3/TniQ) for their enzymatic activities at the PAM-distal end of the DNA^13,14^ (Figure S5C). The kinked nucleotides at positions −1, 6, 12, 18, 24, 30 of the crRNA are stacked with the conserved residues, Trp149s, in the Cas7.1–7.6 subunits, indicating the importance of these periodical interactions in the Cas7 filament-like assembly (Figure S5B). At the end of the Cas7 filament, Cas7.6 binds to a stable Cas5-Cas8 heterodimer, and there is a corresponding minor conformational change from Cas7.5 (r.m.s.d. of 0.62Å for 320 equivalent Cɑ atoms) (Figures S3E and S5D). A Cas5 “left-hand” architecture consisting of the thumb/palm/fingers domains is adjusted for its extensive interactions with elongated Cas8 structural features, such as a long β-hairpin and central/N-terminal domain stacking (Figures S3B, S3D, and S5D). Taken together, the intermolecular interactions of the Cas7 subunits and neighboring subunits along with the crRNA determine the unique ring-like structure of SsCascade.

### DNA targeting by Cascade scaffold

The Cas7.6-bound Cas5-Cas8 heterodimer plays key roles in the DNA-targeting by the RNA-guided surveillance complex. Those three molecules hold the crRNA 5′-handle that is next to the spacer region base-pairing with the DNA target strand (Figures 1C and Figure S5E). The 5′-handle S-shape, consisting of a kinked 5′-end and pseudo-A-formed by the remaining nucleotides, is built by its interactions with conserved structural features, such as the β-hairpin of the Cas8 harpoon, the loop of Cas5 thumb, the first ɑ-helix (ɑ1) of the Cas5 fingers, and the β-sheet of the Cas7.6 fingers^15^ (Figure S5E). The PAM-containing DNA duplex is surrounded by a positively charged vise consisting of the Cas8 N-terminal domain, Cas5 thumb, and Cas7.6 fingers. The N2 of the dG(−2) nucleotide in the target strand is recognized by Asp82_Cas8_, explaining the second C preference in the 5′-NCN-3′ PAM (Figures S6A and S6B). Immediately after the PAM region, the target strand is flipped into the crRNA with the assistance of the Cas8 central and Cas5 thumb domains (Figure S6A). The Ser190_Cas8_/His95_Cas5_ side chains and the Ala94_Cas5_/Ile96_Cas5_ main chains have contacts with the backbone between the dC(−1) and dC1 residues (Figure S6B). The D82A_Cas8_, S190A_Cas8_, H95A_Cas5_, and I96P_Cas5_ mutants reduced the DNA cleavage activity of Cascade, confirming the importance of these target DNA interacting residues (Figure S6C). The A94G_Cas5_ mutant maintained WT-like activity, suggesting the main chain interaction with target DNA, common in alanine and glycine residues, is important for DNA-unwinding. Taken together, the Cas5–Cas8–Cas7.6 functional triad coordinates three molecule events, i) 5′-handle holding, ii) PAM recognition, and iii) target strand displacement, for the subsequent R-loop formation in a conserved manner.

### DNA cleavage by HNH-Cascade machinery

Distinct from other Cascade systems, the SsCascade cleaves a specific site of the target DNA via the uniquely-inserted HNH endonuclease domain (Residues 231–343) in Cas8. The SsCascade HNH, consisting of an anti-parallel β-sheet (β1–2) and surrounding three ɑ-helices (ɑ1–3), folds into a canonical ββɑ-metal structural motif with the conserved functional residues His304_Cas8_, Asn318_Cas8_, and His327_Cas8_ (Figures S3B and S7A). As observed in our previous study, His304_Cas8_, a general base for hydrolysis of phosphodiester bonds, is responsible for genome editing activity in mammalian cells, supporting the importance of this residue for target DNA cleavage^4^. 3D variability analysis showed conformational heterogeneity of the HNH and its association with the head region (Cas6) movement (Movie S1). In density Map A, we observed transient interactions between the HNH and other Cas proteins (Figure 1B). The apical part of the HNH, especially ɑ1, ɑ3, and β1–2, has extensive contacts with the ɑ-helix (ɑ2) in the C-terminal RNA-binding domain of Cas6, while the HNH side and base are supported by the Cas7.1 web and Cas7.6 palm (Figure S7B). The ɑ-helix (Residues 223–230) and resting part (Residues 214–222) of the linker connecting the HNH and central domains are anchored by the Cas7.6 thumb/web and Cas5 thumb in HNH-Cascade-specific and Cascade-wide manners, respectively (Figure S7B). These interactions enable the completion of the positively-charged groove along the HNH-Cascade inner ring accommodating the R-loop/catalytic site and potentially directing the sequential nicking of both DNA strands in a precise manner (Figure S7C).

**Figure 2.**
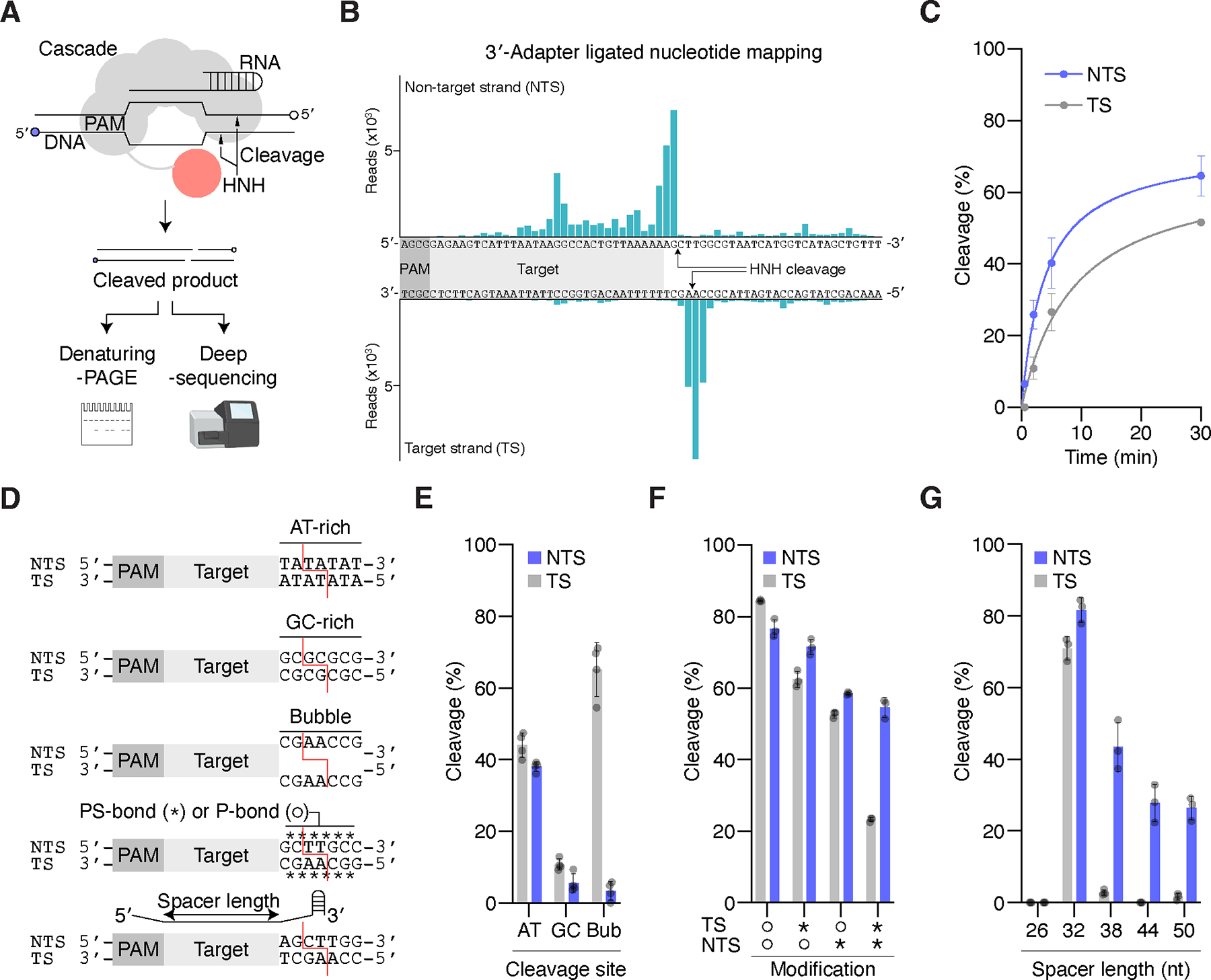
DNA cleavage profile of SsCascade (A) Schematic of in vitro cleavage assay. In all cleavage experiments, the GCG PAM-containing dsDNA substrate was incubated with SsCascade at 37°C for 30 min, unless otherwise indicated. (B) Cleavage products generated by SsCascade. (C) Kinetics of target strand (TS) and non-target strand (NTS) DNA cleavage by SsCascade. The substrate was incubated with SsCascade for 0.5, 2, 5, and 30 min. Error bars represent s.d. from n = 3 (D) Schematics of engineered DNA substrates and crRNA. The red lines indicated the predicted preferred cleavage site. PS-bond, phosphorothioate bond; P-bond, phosphate bond. Error bars represent s.d. from n = 3. (E–G) In vitro DNA cleavage or nicking activities of SsCascade. In (E), the substrates contain the CCG PAM and AT-rich (AT)/GC-rich (GC)/bubble (Bub) sequences in the PAM distal regions. In (F), the substrates contain phosphorothioate modifications (indicated by *) in the TS or NTS. In (G), the substrate was incubated with SsCascade containing different spacer lengths (26, 32, 38, 44, or 50 nt) of crRNAs for 10 min. See also Figures S4 -S6. Error bars represent s.d. from n = 3 (F and G) or n = 4 (E).

To gain further insights into how the HNH domain cleaves both DNA strands, we examined the Cascade-mediated cleavage site and efficiency (Figure 2A). Deep-sequencing analysis showed that Cascade most efficiently cleaved the target strand (TS) and non-target strand (NTS) 37- and 35-bp downstream of the PAM, respectively, (Figure 2B), consistent with previous Sanger-sequencing data^4^. Furthermore, we observed suboptimal cleavage sites, such as 36-/38- and 34-/19-bp downstream the PAM in the TS and NTS, indicating the flexibility of the HNH cleavage site determination. In terms of efficiency, NTS cleavage occurred slightly faster than TS cleavage (Figures 2C and S8A). To test if TS cleavage requires prior NTS cleavage, we conducted an in vitro cleavage assay with various modified DNA substrates (Figures 2D–F and S8B–C). Efficient TS cleavage occurred even in the presence of the bubble-containing DNA substrate, which leads to markedly reduced NTS cleavage, indicating that TS cleavage is independent of NTS cleavage (Figures 2E and S8B). In addition, the substrate containing an AT-rich sequence in the PAM-distal region, which is more likely to be unwound than GC-rich sequence, was more efficiently cleaved than the substrate with a GC-rich sequence in the same region, indicating that TS cleavage likely occurs when the DNA is single-stranded. Based on these results, it seems the mechanism of DNA cleavage is likely determined by complex rules, with clear evidence of interdependence between NTS and TS cleavage, which can allow the same HNH-Cascade system to function as a dsDNA nuclease or a TS or NTS nickase depending on the precise target and guide RNA configurations.

To understand the functional cooperation of the HNH and uniquely large R-loop in the CRISPR-Cas system, we examined the cleavage activities with different R-loop forming conditions (Figures 2G and S9). Given that (i) six Cas7 molecules periodically bind to every 6-nt guide segment in SsCascade, (ii) the regularly-extended crRNA maintains the function of RNA-guided DNA targeting complex in PaCascade, and (iii) the crRNA length defines the number of Cas7 molecules recruited in another Cascade derived from *Shewanella putrefaciens*, we hypothesized that the regularly-extended crRNA recruits greater than six Cas7 molecules and forms a larger R-loop during SsCascade-mediated DNA targeting and cleavage^16–18^. Whereas the crRNA with 26-nt guide segment (hereafter called crRNA26) abolished the DNA cleavage activity of SsCascade, crRNA32 (the natural guide length) induced Cascade-mediated DNA cleavage (Figures 2G and S9B). crRNA38/44/50 (extended guide lengths) efficiently induced Cascade-mediated DNA nicking of the NTS, but not the TS. The most efficient nicking sites are shifted towards the PAM (29/22/23-nt downstream of the PAM with crRNA38/44/50) (Figure S9C). These results indicate that the NTS, which is a flexible part of the R-loop, can access the HNH active site, but that the TS, which is accommodated in the Cascade machinery, may have limited accessibility to the HNH active site, potentially due to the steric hindrance of one or more extra Cas7 molecules (Figure S8D). Furthermore, the native ring-like structure with crRNA32 base-paired its target DNA facilitates the efficient and precise DNA cleavage of Cascade. Taken together, the structural and biochemical data suggest that the conserved HNH domain inserted into Cascade contributes to TS and NTS DNA cleavage in an asymmetric manner.

### Cas8 is a platform for modularity in Cascade systems

Although various type I-F Cascades (SsCascade, PaCascade, and VcCascade) have similar DNA-binding scaffold structures and compositions, such as Cas6, Cas7.1–7.6, and Cas8-Cas5 fusion protein/heterodimer, the C-terminal domain of Cas8 (hereafter called CTD_Cas8_) provides a platform to create functionally diverse Cascade systems. Whereas the SsCascade CTD_Cas8_ HNH works without auxiliary proteins, the PaCascade and VcCascade CTDs_Cas8_ physically contact Cas3 and TniQ for target DNA degradation and insertion, respectively^13,14^ (Figure S10A). To examine the importance of CTD_Cas8_ in DNA targeting, we fused a synthetic transcriptional activator, VPR, to each Cas7, and measured the resulting expression levels of a target gene (Figure S10B). Although both SsCascade and PaCascade induced target gene activation, it was much lower in the absence of CTD_Cas8_(Figure S10C). EMSAs testing the direct binding of Cascade to target DNA did not show reduced binding when the CTDs_Cas8_ was deleted, however, indicating additional features of Cascade are involved in DNA binding (Figure S10D). These results suggest that minimally functional Cascade scaffolds (RNA-guided surveillance complexes) have diverse functions, which are further complemented by their unique CTD_Cas8_ modules.

## Discussion

In this study, we showed that a common Cascade scaffold, a PAM-dependent, RNA-guided DNA targeting complex, has adapted to work with a unique insertion, an HNH endonuclease, creating a ring-like ribonucleoprotein architecture (Figure 1C). Previously, we examined the genome-wide DNA cleavage specificity of SsCascade and did not identify any off-target activity in human cells^4^. This remarkable specificity, defined both by DNA binding and cleavage activities, may be related to the structural features of this complex. Given that the conserved Cas7 Trp149s flip the crRNA nucleotides at the 6th, 12th, 18th, 24th, and 30th positions in both the PaCascade and SsCascade structures (Figure S5B), and that mismatches in these positions are allowed for DNA targeting in PaCascade, these mismatches may be tolerated for DNA cleavage in SsCascade, suggesting the target specificity is defined by the other 27 base-pairings between the crRNA and DNA, as well as the PAM^16^. Another important specificity determinant for target cleavage may be the dynamic interactions between the HNH and other Cas proteins and the ring-like overall structure completed by the HNH domain (Figure S7B, Movie S1), consistent with the biochemical observation that only a crRNA32-loaded Cascade cleaves dsDNA (Figure 2). This hypothesis of HNH cleavage regulation by the Cascade ring structure and its conformational checkpoint will be corroborated by resolving the HNH-Cascade R-loop structure.

**Figure 3.**
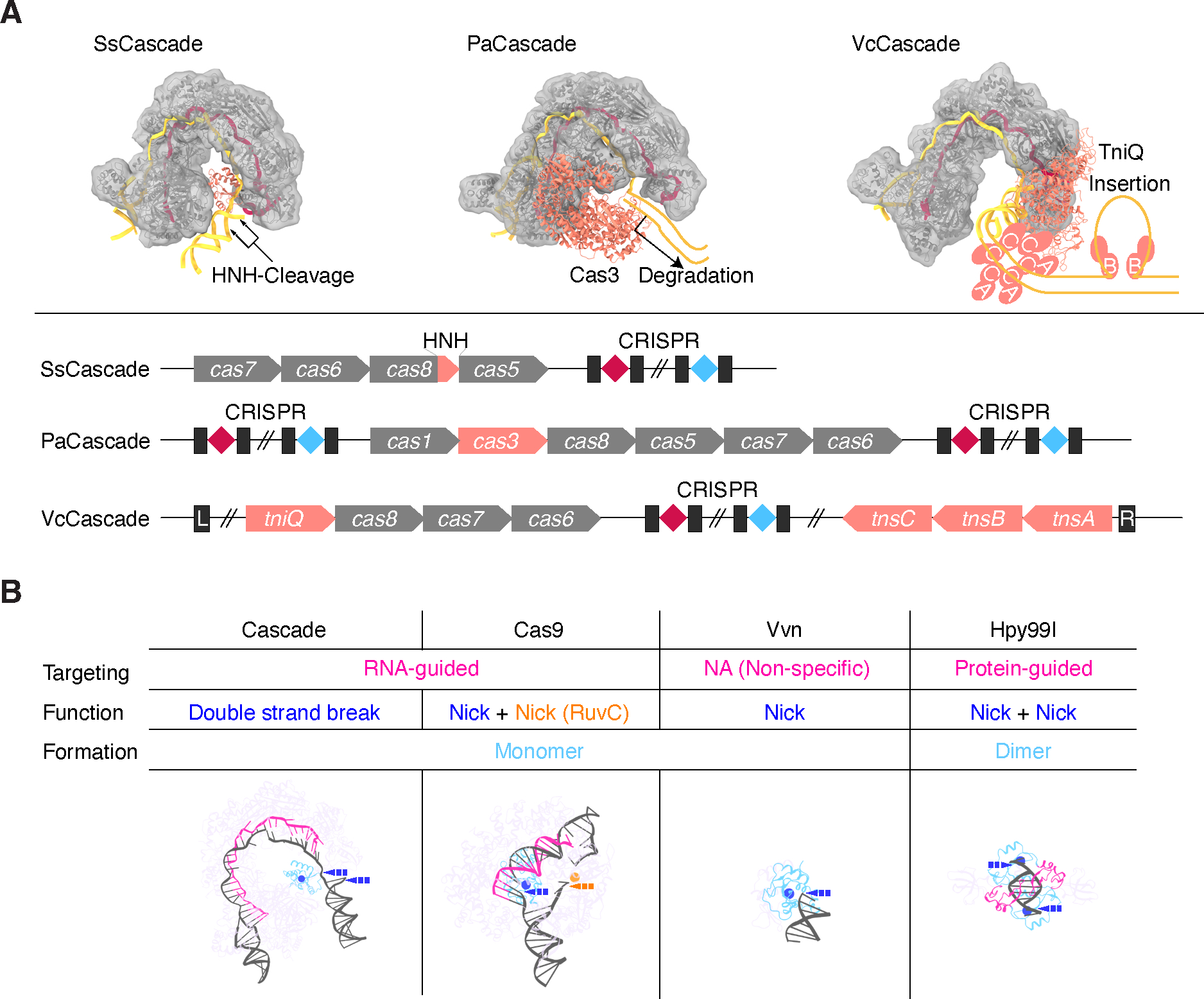
HNH-Cascade is a hybrid of two variable components (A) The type I-F CRISPR-Cas effectors achieve different enzymatic functions through variation in the Cas8 subunit. Molecular and locus architectures of SsCascade, PaCascade (PDB 6NE0), and VcCascade (PDB 6PIJ) are shown. Cascade modularly interacts with functional domains (HNH), other Cas subunits (Cas3), or other machinery (Tn7-like systems consisting of TniQ and TnsA/B/C), highlighted in orange. The target DNA duplex in the PAM-distal region was added to each Cascade docking model, based on the Cas7.1 superposition with another type I-F Cascade structure (PDB 7U5D). PaCas3 was added to the PaCascade model, based on the Cas8 C-terminal domain superposition with a similar protein, AcrF3, bound to PaCas3 (PDB 5B7I). Blurred maps are generated from atomic coordinates by the molmap program and added to the model to highlight the Cascade backbone shapes. (B) HNH nucleases operate in a variety of mechanistically distinct modes. Cascade, Cas9 (PDB 7S4X), Vvn (PDB 1OUP), and Hpy99I (PDB 3GOX) were selected as representatives of four of these modes. The HNH domains and the catalytic histidine residues in the representatives are colored in light blue and blue, respectively. The target binding RNA/protein parts are colored in pink. The cleavage/nicking target sites are indicated by the arrows. See also Figures S10–S11.

The type I CRISPR-Cas Cascade is highly complex, requiring 3–5 different proteins and assembling 8–12 molecules for RNA-guided DNA targeting^17,19^. The main (trunk) part of the Cascade architecture is defined by the crRNA and its spacer length, because Cas7 grips every 6 nt of the RNA for its filament assembly. This relationship can be seen in existing Cascade structures: type I-E/F effectors accommodate crRNA32, whereas the type I-B effector, which has a distinct Cas6-Cas7.1 interface, accommodates crRNA37^19^, and the type I-C effector, which lacks Cas6 and recruits one more Cas7 subunit, accommodates crRNA35^20^. Among these Cascades, the type I-F effector shows the tightest helical pitch, serving as a bridge between two molecular events—PAM recognition and Cascade-related enzymatic activity on the target DNA (Figures 3A and S5C). This connection is achieved either by Cas8 itself or Cas8 in association with other proteins like Cas3 or Tn7-like machinery. This modularity provides an evolutionary platform for generation of diverse defense/transposon systems^21,22^. In the HNH-Cascade system, in the presence of crRNA32, but not crRNA26/38/44/50 (Figure 2), Cas8 has acquired an HNH domain, leading to the functionality to nick target DNA on both strands. This molecular architecture, in which an HNH domain bridges DNA cleavage in Cascade, may be shared by other type I CRISPR-Cas systems, such as the type I-E Cas5-HNH system, which may analogously adopt a “seahorse”-shape with a tail (Cas5-Cas8 heterodimer)-anchored HNH domain and may use a similar mechanism for dsDNA cleavage ^4,7,8^. The flexibility to use a longer crRNA provides a straightforward path to turning the system into a NTS nickase, which could be useful in the prime editing context.

HNH nucleases are found throughout all kingdoms of life and involved in divergent biological functions^23^, such as bacterial defense (e.g., Vvn, Hpy99I, Cas8, and Cas9 for viral DNA interference), transposable element mobility, phage life cycle (e.g., Endo VII for DNA packaging), and genome evolution (e.g., I-PpoI for intron homing) (Figures 3B and S11A). This diversity is reflected in the variations of HNH nucleases, which are adapted to these mechanistically divergent systems. In bacterial defense systems, for example, type II restriction endonucleases, such as Hpy99I, require only additional DNA-binding domains/motifs in the HNH-containing protein for viral DNA recognition^24^. By contrast, Cas effectors, such as Cas8-HNH (HNH-Cascade), Cas5-HNH, DinG-HNH, and Cas9, rely on both guide RNAs and PAM-interacting domains/motifs to target DNA^4,6^. Moreover, although HNH-Cascade introduces DNA nicks in both target DNA strands, in Cas9, the HNH domain induces DNA nicking in only the target strand while a RuvC domain completes the double-strand break^25^. A structural comparison between HNH-Cascade and Cas9 illuminates the molecular adaptation of the HNH domain in CRISPR-Cas systems (Figure S11B). The HNH domain in HNH-Cascade forms parts of a larger ring-like structure, whereas in Cas9 it is part of a bilobed architecture. In HNH-Cascade, this domain contacts the Cas6 subunit and is connected to the rest of Cas8 with a linker that is far from the DNA unwinding point. By contrast, in Cas9, HNH contacts the REC lobe and is inserted in the RuvC domain with two linkers that are close to the DNA unwinding point. These differences in HNH position and contacts give rise to either versatile (HNH-Cascade) or fixed (Cas9) cleavage positions.

Another key difference between HNH-domain containing proteins is their formation: The HNH in Cascade works as a monomer, relying on RNA guiding and flexible HNH tethering to achieve targeted DNA cleavage, whereas other HNHs, such as Endo VII and I-PpoI, work as dimers for targeting holiday junction structures or long (≥14-bp) sequence motifs, which are then doubly nicked^26,27^. Our work reveals how a newly described defense system arising from capture of an HNH domain by a Cascade complex gives rise to a system that precisely creates two DNA nicks, orchestrated by extensive RNA-DNA base-pairings within a ribonucleoprotein ring. This work highlights the modularity of Cascade complexes as a platform for evolutionary novelty as well as the adaptability of HNH nuclease domains for achieving diverse functions.

### Limitations of the study

We note that despite several attempts, we were unable to resolve the full R-loop structure, a model of which would advance our understanding of the mechanism of HNH cleavage. This region may be challenging to resolve due to the dynamic DNA cleavage mechanism of HNH-Cascade and HNH-Cascade’s correspondingly large, flexible R-loop (32 bp), which is likely not conducive to single-state structure determination. This is reflective of the general nature R-loops in CRISPR systems: among 583 CRISPR structures (RNA and DNA-associated complexes) in the Protein Data Bank, to our knowledge, there is only one structure containing clearly visible density of a complete, intact R-loop (17 bp), namely Cas12m, in which its species-specific domain binds the non-target strand extensively, likely stabilizing the loop (PDB: 8HHL)^28^.

## STAR Methods

### Resource Availability

#### Lead contact

Further information and requests for resources and reagents should be directed to and will be fulfilled by the Lead Contact, Feng Zhang (zhang@broadinstitute.org)

#### Materials availability

Plasmids generated in this study are available from Addgene or by request. All other reagents are available upon request.

#### Data and code availability

- The atomic coordinates have been deposited in the Protein Data Bank. The EM map has been deposited in the Electron Microscopy Data Bank (Key Resources Table).
- This paper does not report original code.
- Any additional information required to reanalyze the data reported in this paper is available from the lead contact upon request.

### Experimental Model and Study Participant Details

#### Cell culture

Protein was expressed in *E. coli* Rosetta(DE3)pLysS cells (Novagen), and the cells were cultured at 37°C in autoinduction terrific broth (TB) medium supplemented with 100 mg/L ampicillin and 100 mg/L kanamycin until reaching OD600 of 0.5, then shifted to 18°C for overnight induction. For protein expression in human cells, each component (e.g., Cas5/6/7/8) (*E. coli* codon optimized sequence) was cloned into a CMV promoter-based vector, as described previously^4^. The cognate CRISPR mini array was cloned into a U6 promoter-based vector. HEK293FT cells were cultured in Dulbecco’s modified Eagle medium with high glucose, sodium pyruvate, and GlutaMAX (Thermo), 1× penicillin–streptomycin (Thermo), and 10% fetal bovine serum (Seradigm). Cells were maintained at a confluency below 90%.

### Method Details

#### Electron microscopy sample preparation

Cascade (Cas8-HNH) was expressed in *E. coli* cells, as described previously^4^. Briefly, the *E. coli* codon optimized operon encoding Cas5/6/7/8 proteins was cloned into a pET45b(+) backbone with a His-tag on the Cas8 N-terminus (Addgene Plasmid #205967). The CRISPR mini array containing the direct repeat (5′-GTGTACCGCCGGATAGGCGGTTTAGAAG-3′), reprogrammed spacer (5′-GAGAAGTCATTTAATAAGGCCACTGTTAAAAA-3′), and a second direct repeat, was cloned into a T7 promoter-based vector. These two vectors were co-transformed into *E. coli* Rosetta(DE3)pLysS cells (Novagen), and the cells were cultured at 37°C in autoinduction terrific broth (TB) medium supplemented with 100 mg/L ampicillin and 100 mg/L kanamycin until reaching OD600 of 0.5, then shifted to 18°C for overnight induction. The cells were resuspended in buffer A (50 mM Tris-HCl, pH 8.0, 20 mM imidazole, and 1 M NaCl), lysed by sonication, and then centrifuged. The supernatant was mixed with Ni-NTA Agarose (QIAGEN). The protein-bound column was washed with buffer A and buffer B (50 mM Tris-HCl, pH 8.0, 20 mM imidazole, and 0.3 M NaCl). The SsCascade RNP complex was eluted with buffer C (50 mM Tris-HCl, pH 8.0, 0.3 M imidazole, and 0.3 M NaCl), and further purified by gel filtration chromatography on a Superdex 200 Increase 10/300 column (Cytiva) equilibrated with buffer D (50 mM Tris-HCl, pH 8.0 and 0.5 M NaCl). The SsCascade complex (final concentration: ~4 μM) was mixed with its target DNA consisting of the target (5′-GCAATCAGCTGTTGCTTTTTAACAGTGGCCTTATTAAATGACTTCTCCGTACGCTTGCTGCAACTC-3′) and non-target (5′-GAGTTGCAGCAAGCGTACGGAGAAGTCATTTAATAAGGCCACTGTTAAAAAGCAACAGCTGATTGC-3′) strands (final concentration: 10 μM) at ambient temperature for 5 minutes. For the grid preparation, the Cascade–DNA complex solution (~0.4 μM, 3 μl) was applied to freshly glow-discharged UltrAuFoil 300 mesh R1.2/1.3 grids (Quantifoil) in a Vitrobot Mark IV (FEI) at 6°C with a waiting time of 10 seconds and a blotting time of 4 seconds under 95% humidity.

#### Electron microscopy data collection and processing

Cryo-EM data was collected at the New York Structural Biology Center using EF-Krios, a Titan Krios microscope (Thermo), operated at 300 kV and equipped with a BioQuantum imaging filter (Gatan) and a post-filter K3 direct electron detector (Gatan) in the electron counting mode. A total of 19,805 micrographs were collected using Leginon^29^ v3.5 at 81,000× magnification (1.058 Å per pixel) using image shift, with the nominal defocus from −0.8 μm to −2.5 μm. Each movie was collected with 2.5 seconds of exposure with a total dose of 52.32 electrons per Å^2^, fractionated into 50 frames. To obtain the 3D reconstruction, the data were processed using CryoSPARC v4.3.0, unless otherwise stated^30^. Both global and local motions in the movie frames were corrected by Patch Motion Correction. Defocus parameters were estimated by CTFFIND4^31^. From the 19,805 preprocessed micrographs, 3,644,295 particles were picked by Topaz-based auto-picking^32^ with a trained model from a small batch, and extracted in 4.408Å pixel^−1^. Two rounds of 2D classification were followed to select target particles that had appearances similar to those of typical Cascade complexes. The selected 1,097,058 particles were then re-extracted in 1.202Å pixel^−1^ and subjected to Non-uniform refinement^33^ and 3D classification without alignment^34^ (RELION-4.0). The selected 283,137 particles were subjected to Non-uniform refinement, and yielded a map with a global resolution of 3.28 Å according to the Fourier shell correlation = 0.143 criterion (Map B). This particle population was used for 3D Variability Analysis^35^ (3DVA) to find the Cascade discrete conformations (Movie S1). Apart from the 3DVA, to improve the map quality of the Cascade head part, we created a mask containing Cas7.1 and Cas6 subunits, and used the mask for 3D classification without alignment (RELION-4.0). The selected 80,908 particles were subjected to Non-uniform refinement with per-particle defocus and per-group CTF parameter optimization, as well as spherical aberration, tetrafoil, and anisotropic magnification fitting. The final map yielded a global resolution of 3.48 Å according to the Fourier shell correlation = 0.143 criterion (Map A).

#### Model building and validation

The initial model was generated using ModelAngelo^36^. Part of the initial protein model was replaced with other models generated by AlphaFold2 under the ColabFold framework using default parameters and MMseqs2 to search for homologs into the ColabFold database^37,38^. Part of the initial nucleic acid model was replaced with other structural models of PaCascade-bound nucleic acids (PDB IDs: 6B44 and 2XLI). This chimeric initial model was manually modified using COOT and ISOLDE against the density map, and refined with phenix.real_space_refine, to produce the final Cascade-target DNA complex model^39–41^. Molecular graphics and EM-density figures were prepared with CueMol (http://www.cuemol.org) or Chimera X (https://www.cgl.ucsf.edu/chimerax/).

#### *In vitro* RNA processing experiments

The pre-crRNA substrate was transcribed from the same CRISPR mini array as was used in the cryo-EM sample preparation by T7 Quick High Yield RNA Synthesis kit (NEB), 3′-labeled by pCp-Cy5 (Jena Bioscience) and T4 RNA ligase 1 (NEB), and purified using an RNA Clean and Concentrator kit (Zymo). The pre-crRNA substrate was processed by Cas6, which was expressed using a PURExpress In Vitro Protein Synthesis kit (NEB), at 37°C for 45 minutes, and then resolved on a Novex 10% TBE-Urea gel (Invitrogen) and visualized using a ChemiDoc Imaging System (Bio-Rad).

#### *In vitro* DNA cleavage experiments

The Cascade complexes containing crRNA26/32/38/44/50 were prepared using the same method as described above. The diameters of these complexes were measured by dynamic light scattering, using a DynaPro NanoStar (Wyatt Technology). 145-bp PCR-amplicon (final concentration: ~9 nM) containing the target sequence and the 5′-GCG-3′ PAM was fluorescently labeled with 5′IRDye700/800 (IDT) for the target/non-target strands and then incubated with the Cascade complex (final concentration: ~40 nM) in Buffer E (20 mM Tris-HCl, pH 8.0, 50 mM NaCl, and 2.5 mM MgCl_2_) at 37°C for 30 minutes, resolved on a Novex 10% TBE-Urea gel, and visualized using a ChemiDoc Imaging System. The cleavage products were quantified, using GelAnalyzer (http://www.gelanalyzer.com/). The cleavage kinetics were fitted into the Michaelis-Menten model. DNA substrate containing phosphorothioate modification was prepared from purchased oligonucleotides (IDT).

#### Genome editing assay

For genome editing analysis, 96-well plates were seeded with 15,000 cells/well 16 hours before transfection. Cells were transfected with 50 ng of each Cascade component (Cas5/6/7/8 and crRNA) in Opti-MEM (Thermo) with 0.75 μl TransIT-LT1 transfection reagent (Mirus). Cells were harvested 72 hours post-transfection, and genomic DNA was extracted by QuickExtract DNA Extraction Solution (Biosearch technologies). The target genomic region was amplified with Phusion Flash HF-PCR Master Mix (Thermo), and then Illumina adapters and barcodes were added. The library was gel extracted and subjected to single-end sequencing on a MiSeq System (Illumina). Insertion/deletion (indel) frequency was calculated using CRISPResso2 with quantification window size_4/center_3 flags^42^.

#### Transcription activation assay

For the transcription activation experiment, the VP64-p65-Rta (VPR) (Addgene Plasmid #63978) was appended to the Cas7 C-terminus. The Cas8 C-terminal regions (Residues 231–344 [SsCas8] and 277–434 [PaCas8]) were deleted for mutants. The sequence (5′-CTGCCTAAGGATGTGGGGCTGTCAGCAGATCT-3′) upstream the *TTN* gene was targeted for transcriptional activation. 12-well plates were seeded with 150,000 cells/well 16 hours before transfection. Cells were transfected with 100 ng of each Cascade component (Cas5/6/7/8 and crRNA, summarized in Figure S9C) in Opti-MEM with 1.5 μl Lipofectamine3000 and 1 μl P3000 transfection reagents (Thermo). 72 hours after transfection, total RNA was purified from harvested cells by RNeasy (Qiagen), and reverse-transcribed by SuperScript VILO Master Mix. Per qPCR reaction, 5 μl of cDNA was mixed with PerfeCTa SYBR Green FastMix (Quantabio) and qPCR primers (Key Resource Table), and run using CFX Opus 384 Dx System (Bio-Rad). All qPCR data are presented as fold change in target (*TTN*) gene expression normalized to calibration (*GAPDH*) gene expression.

#### Electrophoretic mobility shift assay

The PaCascade protein expression vector was a gift from Blake Wiedenheft (Addgene Plasmid #89232). SsCascade and PaCascade were prepared using the same expression and purification methods as described above. Each Cascade (final concentration: ~150 or 450 nM) was incubated with the GCG PAM containing target DNA (the same substrate as described in the DNA cleavage assay, final concentration: ~5 nM) in Buffer D (20 mM Tris-HCl, pH 8.0, 50 mM NaCl) at 37°C for 10 minutes, resolved on a Novex 6% TBE gel, and visualized using a ChemiDoc Imaging System.

#### Sequencing of cleavage products

149-bp PCR-amplicon containing the target sequence and the GCG PAM was prepared and incubated with SsCascade complexes at 37°C for 30 min. The reactions were subjected to a GLOE-seq library preparation protocol as described in a previous study^42^. The final amplification to add Illumina adapters and barcodes was performed with Phusion Flash High-Fidelity PCR Master Mix (Thermo). Libraries were subjected to paired-end sequencing using an Illumina MiSeq with Read 1 150 cycles, Read 2 150 cycles, Index 1 8 cycles and Index 2 8 cycles. Paired-end reads were mapped to the target substrate and 3′ ends were extracted and plotted using a custom Python script described in our previous study^43^.

### Quantification and Statistical Analysis

Where relevant, the statistical approach for each experiment is described in the figure legend (e.g., standard deviation, student t-test).

## Supplementary Material

Supplementary Materials

Movie S1 **Supplementary Movie S1. Head-to-tail association via interactions between Cas6 and HNH in SsCascade**, related to Figure 1B.
3D Variabity Analysis of SsCascade particles before Cas6-maksed refinement. The ensemble of SsCascade discrete conformations indicates the pinching motion of the SsCascade backbone and its association with Cas6 (part of the SsCascade head)-HNH (part of the SsCascade tail) contact. This analysis was conducted before the Map A reconstruction (Figures 1B and S1C).

## Figures and Tables

**Table 1 |:** Data collection, processing, refinement, and validation statistics

**Data collection and processing**		
Sample	SsCascade	
EMDB ID	43729	
PDB ID	8W1P	
Microscope	Titan Krios	
Detector	Gatan K3	
Pixel Size (Å)	1.058	
Defocus range (μm)	−0.8 to −2.5	
Voltage (kV)	300	
Electron dose (e^−^/Å^2^)	52.32	
Number of movies	19,805	
Final particle images	80,908	
Map resolution (FSC = 0.143) (Å)	3.48	
**Refinement and validation**		
Model resolution (FSC = 0.5) (Å)	3.68 (Masked)	3.86 (Unmasked)
Map CC (around atoms)	0.78	
Map sharpening B-factor (Å)	−90.7	
Model composition		
Protein atoms	43,015	
Nucleic acid atoms	3,369	
Ligand	0	
Mean *B*-factors (Å^2^)		
Protein	78.79	
Nucleic acid	104.40	
Ligand	N/A	
Root mean square deviations		
Bond lengths (Å)	0.006	
Bond angles (°)	0.936	
Validation		
MolProbity score	0.80	
Clashscore	0.32	
Rotamer outlier (%)	0.29	
CaBLAM outlier (%)	1.88	
Ramachandran plot (%)		
Favored	96.93	
Allowed	3.07	
Outlier	0.00	

**Key resources table:** 

REAGENT or RESOURCE	SOURCE	IDENTIFIER
Bacterial and virus strains		
Rosetta^™^ 2(DE3)pLysS Competent Cells E. coli	Novagen	71403
One Shot^™^ Mach1^™^ T1 Phage-Resistant Chemically Competent E. coli	Invitrogen	C862003
Chemicals, peptides, and recombinant proteins		
PrimeSTAR^®^ Max DNA Polymerase	Takara	R045A
KAPA HiFi HotStart ReadyMix	Roche	KK2602
Phusion Flash High-Fidelity PCR Master Mix	Thermo Scientific	F548S
KLD Enzyme Mix	New England Biolabs	M0554S
Novex^™^ TBE-Urea Gels, 15%	ThermoFischer	EC68852BOX
Novex^™^ TBE Gels, 6%	ThermoFischer	EC62655BOX
Invitrogen novex TBE Running Buffer (5X)	ThermoFischer	LC6675
QIAquick PCR Purification Kit	QIAGEN	28106
QIAprep Spin Miniprep Kit	QIAGEN	27106
Gibson Assembly^®^ Master Mix	New England Biolabs	E2611
Isopropyl-β-D-thiogalactopyranoside	Goldbio	I2481C
RNase A	QIAGEN	19101
T4 DNA ligase	New England Biolabs	M0202S
AMPure XP for PCR Purification	Beckman Coulter	A63881
Tn5	Schmid-Burgk et al.^43^	N/A
Dynabeads^™^ MyOne^™^ Streptavidin C1	Invitrogen	65001
UltraPure^™^ SSC, 20X	Invitrogen	15557044
Amicon Ultra-15 Centrifugal Filter Units 10kDa NMWL	Millipore sigma	UFC801024
NuPAGE 4–12% Bis-Tris Protein Gels, 1.0 mm, 12-well	ThermoFischer	NP0322BOX
NuPAGE LDS Sample Buffer (4X)	ThermoFischer	NP0007
Ampicillin, sodium salt	AmericanBio	Ab00115
Kanamycin sulfate from Streptomyces kanamyceticus	Sigma	K4000
Tris (1M), pH 8.0, RNase-free	Invitrogen	AM9855G
Sodium chloride	Sigma-Aldrich	71376
Magnesium chloride hexahydrate	Sigma-Aldrich	M0250
Imidazole, 99%, Thermo Scientific Chemicals	ThermoScientific	A10221.36
Ni-NTA Agarose (25 ml)	QIAGEN	30210
Critical commercial assays		
Qubit 1X dsDNA HS (HighSensitivity) Assay Kit	ThermoFischer	Q33231
eStain L1 Protein Staining System	GenScript	N/A
MiSeq Reagent Kits v2	Illumina	MS-102
NEBNext^®^ Ultra^™^ II DNA Library Prep Kit for Illumina^®^	New England Biolabs	E7645S
Deposited data		
SsCascade micrographs	This work	EMPIAR-12178
SsCascade EM map	This work	EMDB: EMD-43729
SsCascade model	This work	PDB: 8W1P
Experimental models: Cell lines		
293FT Cell Line	Invitrogen	R70007
Oligonucleotides		
CAATGACCCCTTCATTGACC	IDT	GAPDH-FW
TTGATTTTGGAGGGATCTCG	IDT	GAPDH-RV
T GTTGCCACTGGTGCTAAAG	IDT	TTN-FW
ACAGCAGTCTTCTCCGCTTC	IDT	TTN-RV
GCAATCAGCTGTTGCTTTTTAACAGTGGCCTTATTAAATGACTTCTCCGTACGCTTGCTGCAACTC	IDT	Cryo-EM_target-DNA_TS
GAGTTGCAGCAAGCGTACGGAGAAGTCATTTAATAAGGCCACTGTTAAAAAGCAACAGCTGATT GC	IDT	Cryo-EM_target-DNA_NTS
CCTCTGACACATGCAGCTCCATTGAGCGGAGAAGTCATTTAATAAGGCCACTGTTAAAAAG*C*T*T*G*C*CACACAACATACGAGCCGGAAGCA	IDT	Phosphorothioate (*)-incorporated target-DNA_NTS
TGCTTCCGGCTCGTATGTTGTGTG*G*C*A*A*G*CTTTTTAACAGTGGCCTTATTAAATGACTTCTCCGCTCAATGGAGCTGCATGTGTCAGAGG	IDT	Phosphorothioate (*)-incorporated target-DNA_TS
Software and algorithms		
Geneious Prime	Dotmatics	https://www.geneious.com/
Image Lab 1.6	Bio-Rad	https://www.bio-rad.com/
CueMol	Ryuichiro Ishitani	http://www.cuemol.org/en/
ModelAngelo	Jamali et al.^36^	https://github.com/3dem/model-angelo
Prism 10	GraphPad	https://www.graphpad.com/scientific-software/prism/
Leginon 3.5	Suloway et al.^44^	https://emg.nysbc.org/redmine/projects/leginon/wiki/Leginon_Homepage
GelAnalyzer 23.1	Istvan Lazar Jr.	http://www.gelanalyzer.com/?i=1
CTFFIND4	Rohou et al.^31^	https://grigoriefflab.umassmed.edu/ctffind4
Phenix 1.2.1	Liebschner et al.^45^	https://phenix-online.org/
UCSF ChimeraX 1.7	Goddard et al.^46^	https://www.cgl.ucsf.edu/chimerax/
Coot 0.9.8.1	Emsley et al.^39^	https://www2.mrc-lmb.cam.ac.uk/personal/pemsley/coot/
ISOLDE 1.7	Croll et al.^41^	https://tristanic.github.io/isolde/
CryoSPARC 4.3	Punjani et al.^30^	https://cryosparc.com/
Relion 4.0	Kimanius et al.^34^	https://relion.readthedocs.io/
MolProbity	Chen et al.^47^	http://molprobity.biochem.duke.edu/
AlphaFold2	Jumper et al.^37^	https://alphafold.ebi.ac.uk/
ColabFold	Mirdita et al.^38^	https://github.com/sokrypton/ColabFold
CRISPResso2	Clement et al.^42^	https://github.com/pinellolab/CRISPResso2
Adobe Illustrator CC	Adobe	https://www.adobe.com
Other		
Superdex 200 Increase 10/300 GL	Cytiva	28990944
UltrAuFoil R 1.2/1.3, 300 mesh, Gold	Quantifoil^®^	Q350AR13A
