# Supplementary material for: Structural determinants of DNA cleavage by a CRISPR HNH-Cascade system
Source: Mol Cell. Author manuscript; Available in PMC 2024 Oct 8. (PMC11459484; doi:10.1016/j.molcel.2024.07.026)
Supplement: Supplementary Materials [file NIHMS2021639-supplement-Supplementary_Materials.pdf]

Supplementary Figure Legends

Figure S1. Cryo-EM data processing for the SsCascade-target DNA complex, related to Figure 1.
(A) Example cryo-EM micrograph of the SsCascade-target DNA mixture sample.
(B) 2D class averages of the target particles.
(C) 3D reconstitutions in the cryo-EM data processing. The particle population best resolving Cas6 (and HNH consequently) was selected for the final reconstitution.
(D–F) Sharpened/unsharpened maps (D), particle orientation distribution (E), and local resolution estimation (F) in the final reconstitution for Map A. The local resolution was calculated in CryoSPARC.
(G) FSC curves calculated between the half maps from the Non-uniform refinement in CryoSPARC (top). An FSC curve calculated between the model and the final refined map, using phenix.validation_cryoem (bottom).

Figure S2. Cryo-EM density maps, related to Figure 1.
(A–D) Cryo-EM density maps for SsCascade residues represented in entire figures. Panels A–D are related to Figures S4A, S5B, S6B, and S7A, respectively.

Figure S3. Structural details of each Cascade component, related to Figure 1.
(A) crRNA and target DNA with protein components shown (left) or hidden (center) and crRNA schematic (right).
(B–E) Close-up views of the Cas protein structures.

Figure S4. crRNA processing by Cas6, related to Figure 1.
(A) Structure of 3′-hairpin bound to Cas6 in SsCascade. The 3′-phosphate group of the crRNA was derived from the U53 of the continuous direct repeat region and processed by Cas6 endoribonuclease activity.
(B) Structure of PaCas6 bound to 15-nt RNA hairpin (PDB 2XLI). A part (C38–C53) of the PaCascade crRNA was reconstituted with an apo PaCas6 protein and the phosphate group of C53 was processed by PaCas6.
(C) In vitro reconstituted SsCas6 processing of pre-crRNA substrates with wild-type and mutant SsCas6.

Figure S5. crRNA-guided Cas protein assembly in SsCascade, related to Figure 1.
Schematic of crRNA-Cas protein interactions and the corresponding figure panels.
(A) Assembly of the head part of Cascade occurs on the 3′-hairpin of the crRNA in SsCascade (left) and PaCascade (right) (PDB 6B44) structures. In SsCascade, there is only minimal contact between Cas6 and Cas7.1 (as opposed to in PaCascade, which has a second point of contact). This limited interaction directs the head region to the tail region in SsCascade.
(B) Assembly of the trunk part of SsCascade occurs on the spacer region. Interactions between Cas7.4 and the crRNA are similar to those of other Cas7 proteins.
(C) Assembly of the Cas7 filament in the type I-F Cascades (PDB 6B44 [PaCascade] and 6PIJ [VcCascade]). For clarity, only crRNAs and Cas7s are displayed.
(D) Assembly of the tail part of SsCascade occurs on 5′-handle.
(E) 5′-handle recognition by three Cas proteins.

Figure S6. DNA targeting mechanism of SsCascade, related to Figure 1.
(A) PAM-containing DNA duplex bound to the groove of the tail of SsCascade.
(B) PAM recognition and DNA unwinding by the Cas5-Cas8 heterodimer.
(C) SsCascade-mediated indel formation at the *CA2* locus in HEK293FT cells with wild-type and mutant Cascades (mean±s.d.; n = 3 biological replicates). NT, non-target.

Figure S7. HNH domain structure and its functional mechanism in SsCascade, related to Figure 2.
(A) HNH motifs in SsCas8 and SpCas9 (PDB 7S4X). The target DNA is bound to the HNH catalytic site in the SpCas9 structure.
(B) HNH domain interactions with other Cas proteins in SsCascade.
(C) Proposed model of target DNA cleavage within the circular groove of HNH-Cascade (left). The circular groove inside the HNH-Cascade ring is positively charged (right). TS, target strand; NTS, non-target strand.

Figure S8. DNA cleavage mode of wild-type SsCascade with target dsDNA, related to Figure 2.
(A–C) Representative gel images of in vitro DNA cleavage or nicking analysis by SsCascade, related to Figure 2E–G. Primer indicates the primers used in the PCR amplification of the target DNA substrate (which are not removed by column filtration). AT/GC/Bub, Substrates incorporating AT-rich/GC-rich/Bubble into their PAM distal regions; $T^{P}N^{P}$, Substrate without incorporating phosphorothioate modifications; $T^{PS}N^{P}$, Substrate incorporating phosphorothioate modifications into the PAM distal regions in the target strand, but not the non-target strand; $T^{P}N^{PS}$, Substrate incorporating phosphorothioate modifications into the PAM distal regions in the non-target strand, but not the target strand; $T^{PS}N^{PS}$, Substrate incorporating phosphorothioate modifications into the PAM distal regions in both the target and non-target strands.

Figure S9. Distinct DNA cleavage modes of engineered SsCascades with expanded R-loop formations, related to Figure 2.
(A) Size profile of purified SsCascades with different spacer lengths (26/32/38/44/50 nt, referred to as SsCascade26/32/38/44/50), by chromatography and dynamic light scattering. The measured particle diameter of Cascade32 (13.6 nm, DNA-unbound) is consistent with the cryo-EM structure (14.1 nm, DNA-bound).
(B) Representative gel images of in vitro DNA cleavage or nicking analysis by SsCascade, related to Figure 2G.
(C) Sequencing of cleaved or nicked products generated by SsCascades and a restriction enzyme, related to Figure 2B. NTS, non-target strand; TS, target strand; PAM is highlighted in gray. The HindIII (positive control) cleavage site is indicated as a red line. Reads most distal to the PAM on the NTS in the engineered Cascade44 and Cascade50 samples may represent background due to the enlarged R-loop that makes this region susceptible to endogenous nucleases.
(D) Docking models of wild-type (top) and engineered (bottom) crRNA-directed SsCascade bound to target DNA. Compared to the wild-type version (SsCascade32), the engineered version adopts a longer spacer length (SsCascade50), and presumably recruits three more Cas7 protein subunits (Cas7.7–7.9). The PAM-distal DNA duplex was added to each model as described in Figure 3A.

The additional Cas7 proteins were added to the engineered model, based on the Cas7.1 superposition with Cas7.4.

Figure S10. DNA targeting by Cascade, related to Figure 3A.
(A–B) Wild-type (A) and engineered (B) type I-F systems. Engineered Cascades contain the activator VPR fused to Cas7 but lack the Cas8 C-terminal domain (CTD). Complexes were directed via the crRNA to a target site upstream of the *TTN* gene in the human genome.
(C) Gene activation by wild-type or engineered Cascades. Activation was measured by quantitative PCR of the *TTN* gene transcript in HEK293FT cells (mean±s.d.; $n = 3$ biological replicates). The qPCR results of target crRNA transfected conditions are normalized to those of non-target crRNA transfected conditions via ddCt correction. Statistical significance was assessed using a two-tailed *t*-test (*$P < 0.05$, **$P < 0.01$). Cascade components used for each condition are described in the right panel. FL, full-length; WT, wild-type; NT, non-target; Cond.; condition.
(D) Electrophoretic Mobility Shift Assay (EMSA) showing target DNA-binding activities of Cascades (Right). The purified Cascades corresponding to the cellular assay constructs are shown in the left and center panels. Ss, SsCascade; Pa, PaCascade; ΔC, CTD truncation in Cas8; Substrate, target DNA substrate.

Figure S11. Additional mechanistic modes of HNH nucleases, related to Figure 3B.
(A) EndoVII (PDB 2QNC) and I-PpoI (PDB 1A74) are further examples of the mechanistic modes of HNH nucleases in phage DNA packaging and fungus intron homing systems, respectively.
(B) Comparison of HNH-related structural features in CRISPR-Cas systems. In Cascade, the HNH is tethered by a linker (L) to the Cas8 C-terminus. In Cas9, the HNH is inserted into the RuvC domain via two linkers (L1 and L2).

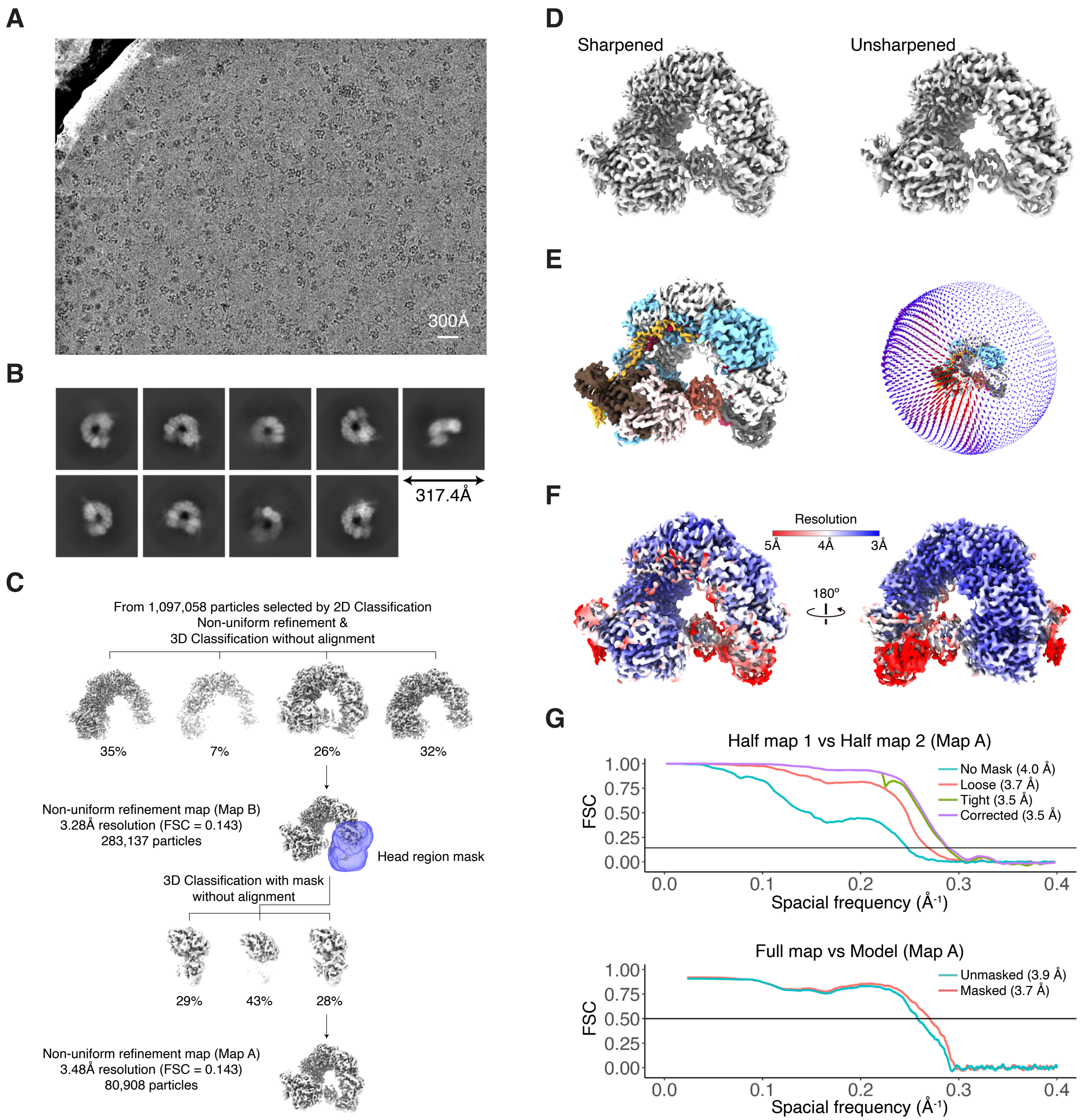
A
300Å
B
317.4Å
C
From 1,097,058 particles selected by 2D Classification
Non-uniform refinement &
3D Classification without alignment
35%
7%
26%
32%
Non-uniform refinement map (Map B)
3.28Å resolution (FSC = 0.143)
283,137 particles
Head region mask
3D Classification with mask
without alignment
29%
43%
28%
Non-uniform refinement map (Map A)
3.48Å resolution (FSC = 0.143)
80,908 particles
D
Sharpened
Unsharpened
E
F
Resolution
5Å
4Å
3Å
180°
G
Half map 1 vs Half map 2 (Map A)
FSC
No Mask (4.0 Å)
Loose (3.7 Å)
Tight (3.5 Å)
Corrected (3.5 Å)
Spacial frequency (Å-1)
Full map vs Model (Map A)
Unmasked (3.9 Å)
Masked (3.7 Å)

Figure S1

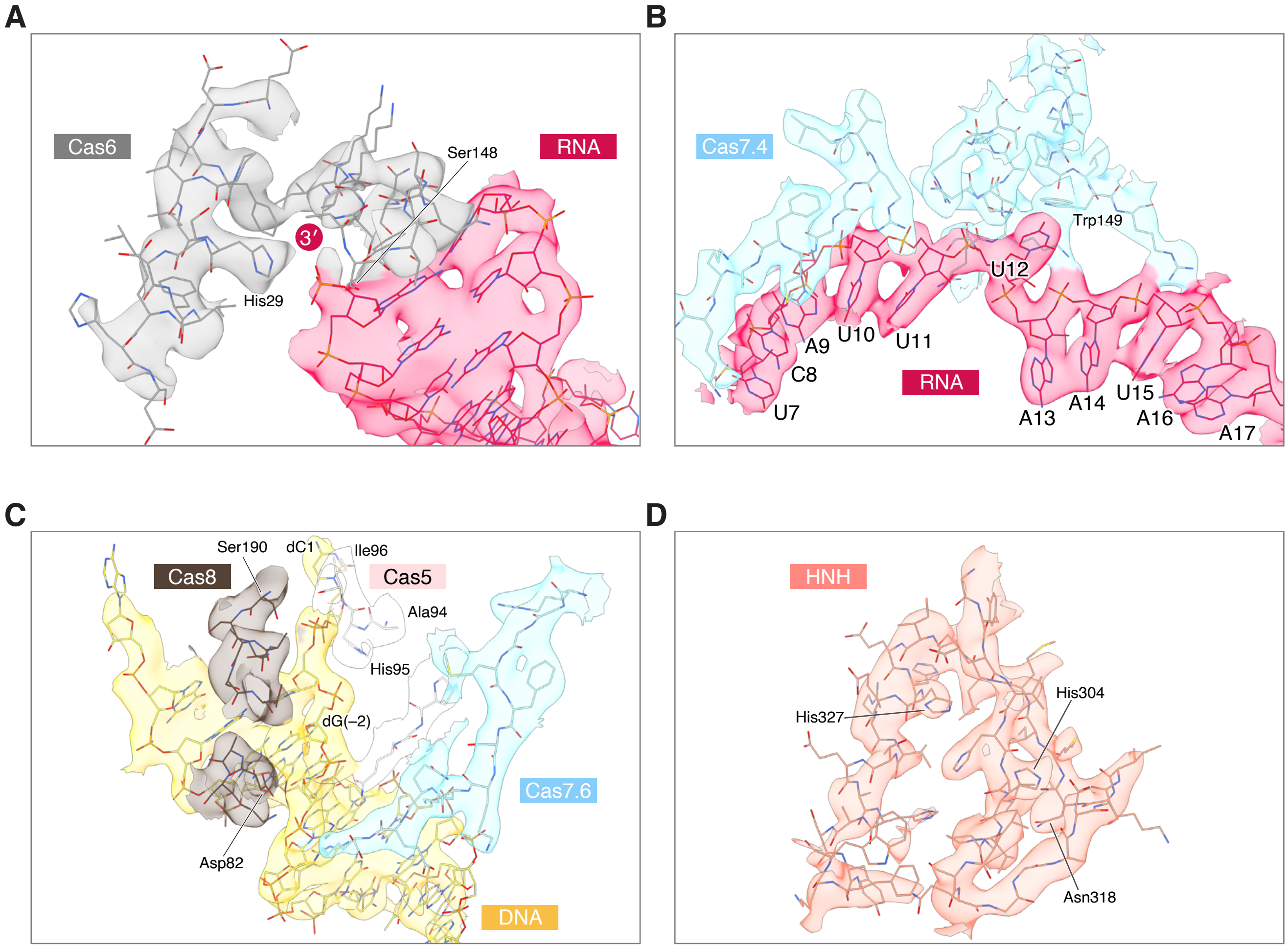

A
Cas6
RNA
Ser148
3′
His29
B
Cas7.4
Trp149
U12
A9
U10
U11
C8
RNA
U7
A13
A14
U15
A16
A17
C
Ser190
dC1
Ile96
Cas8
Cas5
Ala94
His95
dG(−2)
Cas7.6
Asp82
DNA
D
HNH
His327
His304
Asn318

Figure S2

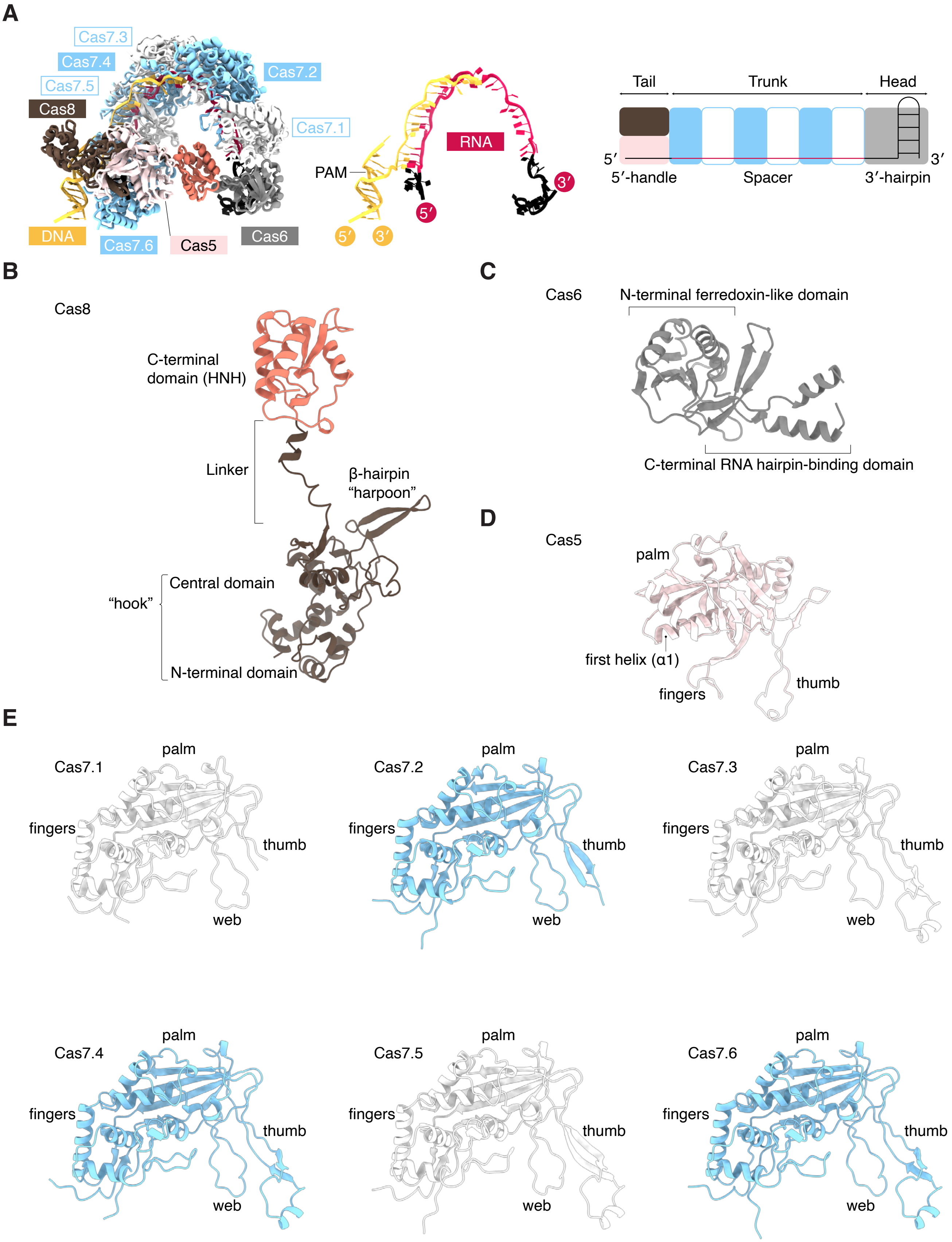
A
Cas7.3
Cas7.4
Cas7.2
Cas7.5
Cas8
Cas7.1
DNA
Cas7.6
Cas5
Cas6
RNA
PAM
5′
3′
5′
3′
Tail
Trunk
Head
5′
3′
5′-handle
Spacer
3′-hairpin
B
Cas8
C-terminal domain (HNH)
Linker
β-hairpin “harpoon”
“hook”
Central domain
N-terminal domain
C
Cas6
N-terminal ferredoxin-like domain
C-terminal RNA hairpin-binding domain
D
Cas5
palm
first helix (α1)
fingers
thumb
E
Cas7.1
Cas7.2
Cas7.3
Cas7.4
Cas7.5
Cas7.6
palm
fingers
thumb
web

Figure S3

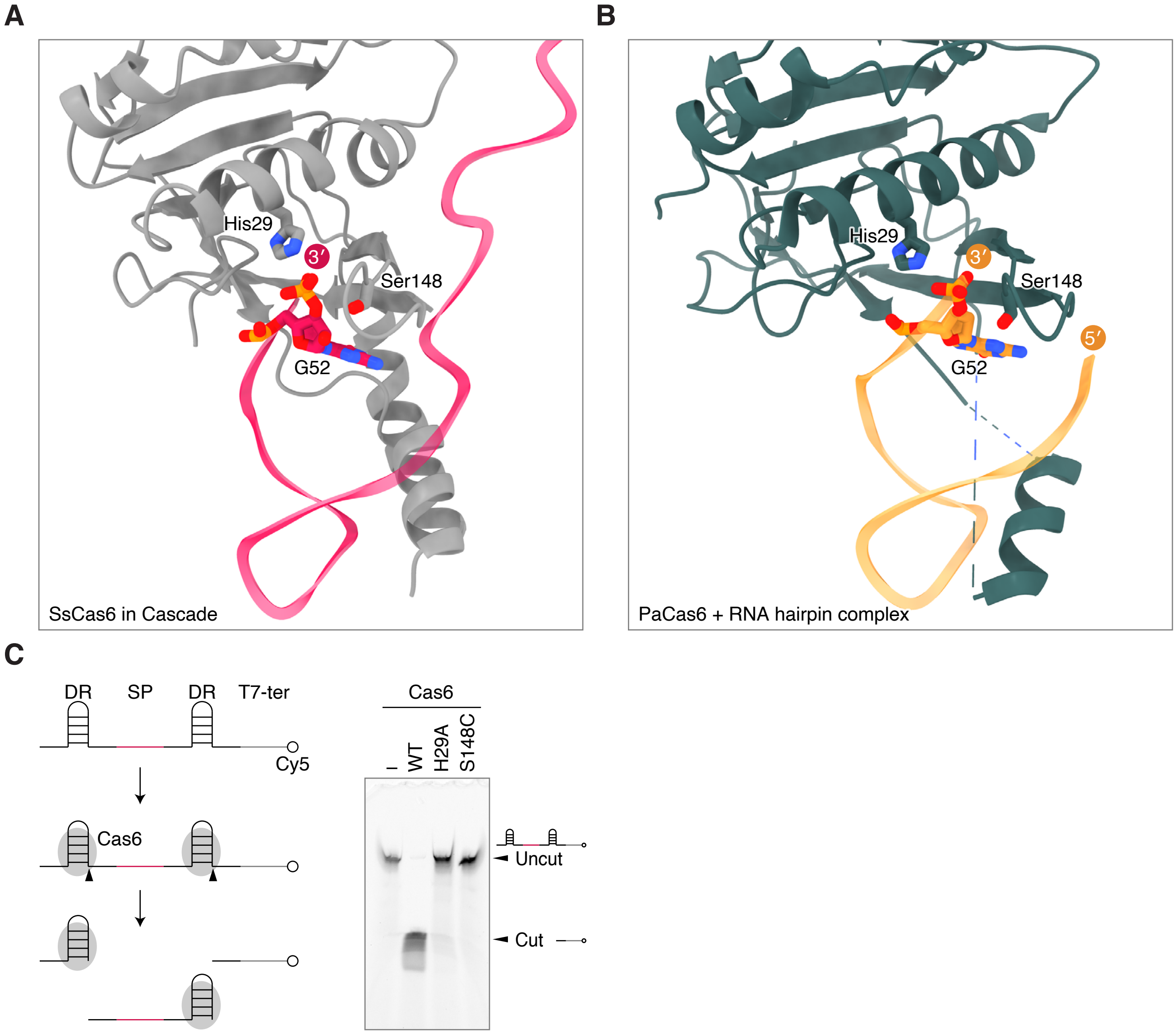
A
His29
3′
Ser148
G52
SsCas6 in Cascade
B
His29
3′
Ser148
5′
G52
PaCas6 + RNA hairpin complex
C
DR
SP
DR
T7-ter
Cy5
Cas6
Cas6
–
WT
H29A
S148C
Uncut
Cut

Figure S4

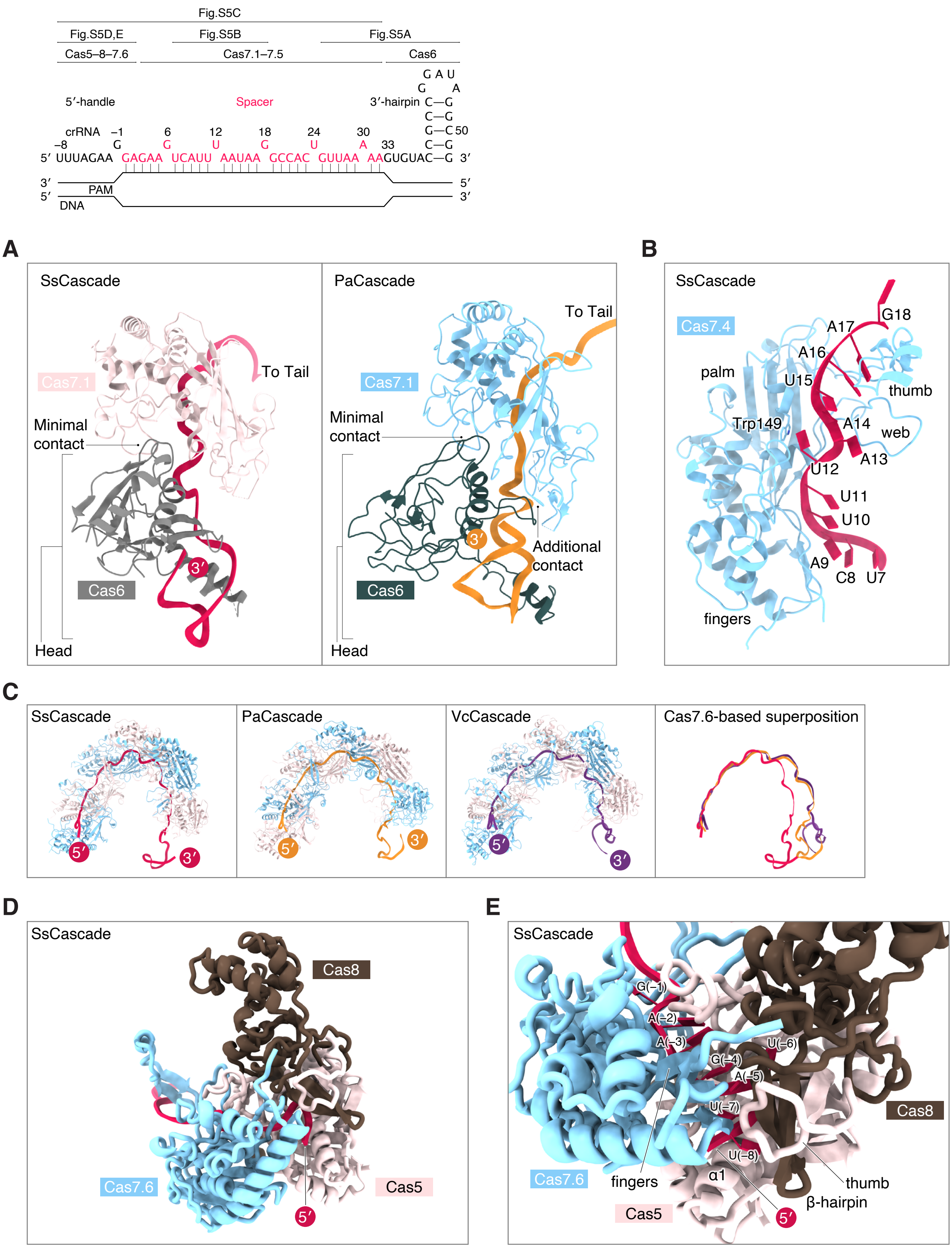
Fig.S5C
Fig.S5D,E
Fig.S5B
Fig.S5A
Cas5–8–7.6
Cas7.1–7.5
Cas6
5′-handle
Spacer
3′-hairpin
crRNA
5′ UUUAGAA GAGAA UCAUU AAUAA GCCAC GUUAA AAGUGUAC—G 3′
PAM
DNA
A
SsCascade
Cas7.1
To Tail
Minimal contact
Cas6
Head
PaCascade
Additional contact
B
Cas7.4
palm
thumb
web
Trp149
fingers
C
VcCascade
Cas7.6-based superposition
D
Cas8
Cas7.6
Cas5
E
α1
β-hairpin

Figure S5

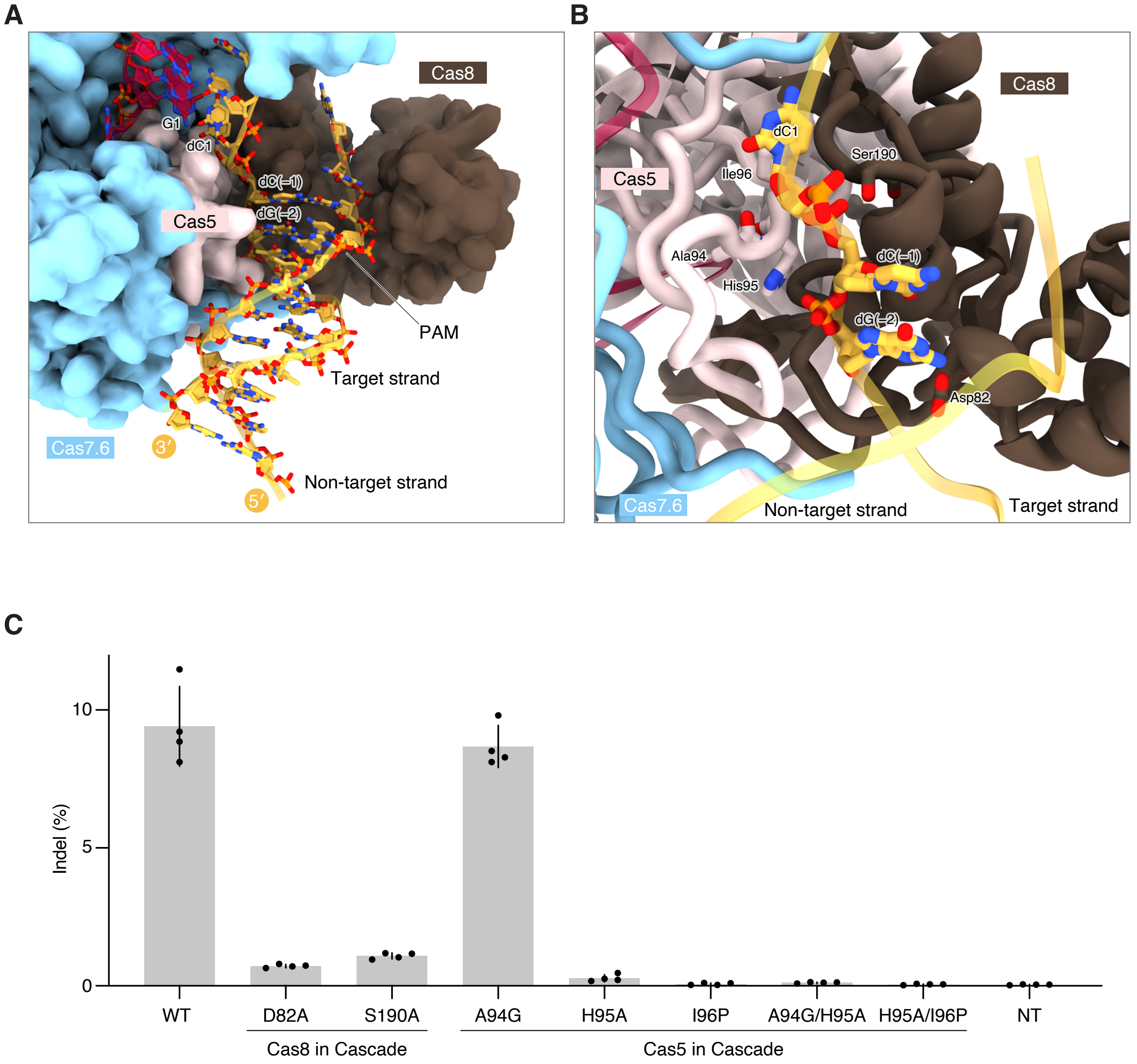
A
Cas8
G1
dC1
dC(−1)
dG(−2)
Cas5
PAM
Target strand
Cas7.6
3′
5′
Non-target strand
B
Cas8
dC1
Ser190
Cas5
Ile96
Ala94
dC(−1)
His95
dG(−2)
Asp82
Cas7.6
Non-target strand
Target strand
C
10
5
0
Indel (%)
WT
D82A
S190A
A94G
H95A
I96P
A94G/H95A
H95A/I96P
NT
Cas8 in Cascade
Cas5 in Cascade

Figure S6

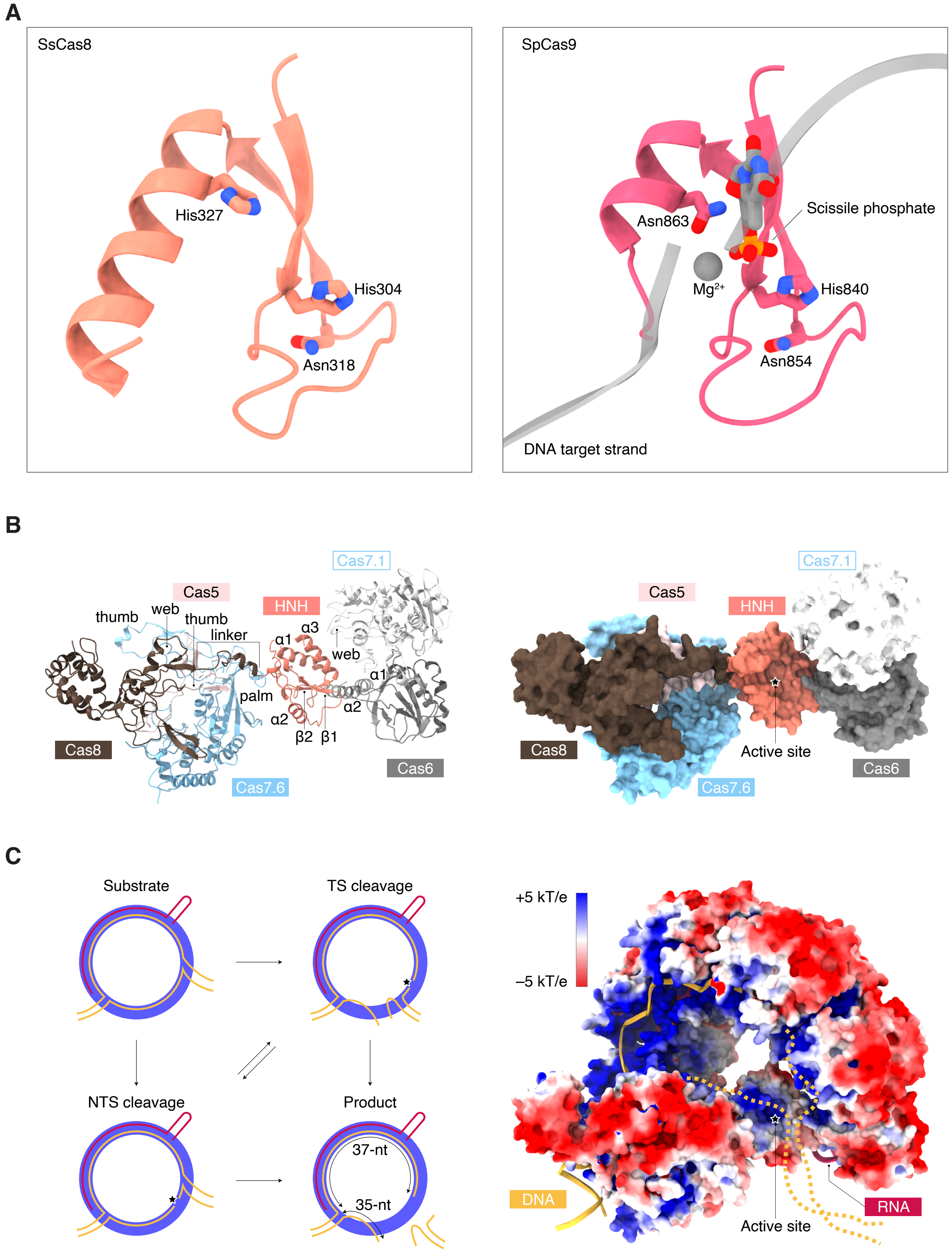
A
SsCas8
His327
His304
Asn318
SpCas9
Scissile phosphate
Asn863
Mg2+
His840
Asn854
DNA target strand
B
Cas7.1
Cas5
HNH
thumb
web
thumb
linker
α1
α3
web
α1
palm
α2
α2
β2
β1
Cas8
Cas7.6
Cas6
Active site
C
Substrate
TS cleavage
NTS cleavage
Product
37-nt
35-nt
+5 kT/e
–5 kT/e
DNA
RNA
Active site

Figure S7

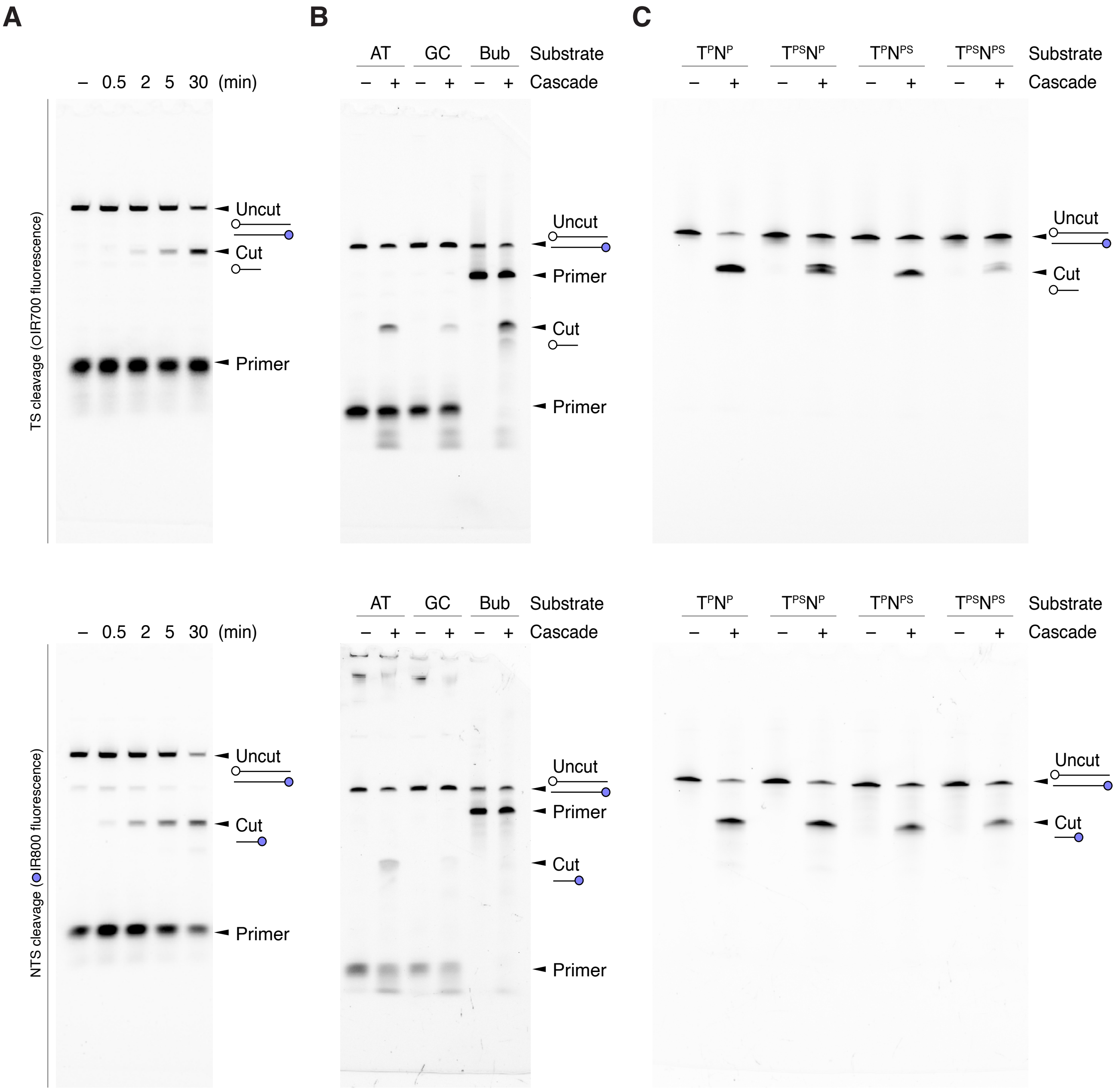

A
B
C
– 0.5 2 5 30 (min)
TS cleavage (IR700 fluorescence)
NTS cleavage (IR800 fluorescence)
Uncut
Cut
Primer
AT
GC
Bub
Substrate
Cascade
– + – + – +
T^P N^P
T^PS N^P
T^P N^PS
T^PS N^PS
– + – + – + – +

Figure S8

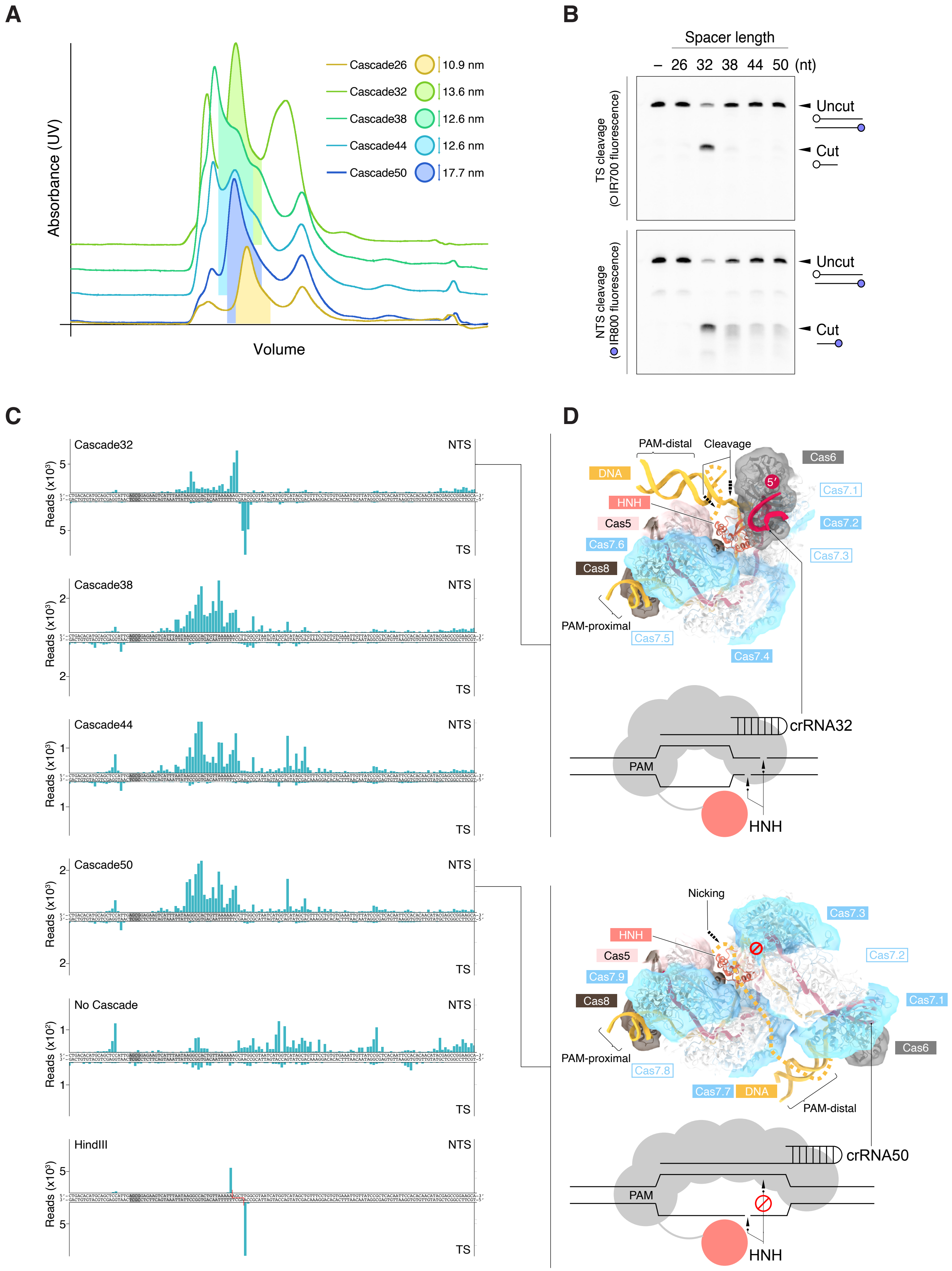
A
Absorbance (UV)
Volume
Cascade26 10.9 nm
Cascade32 13.6 nm
Cascade38 12.6 nm
Cascade44 12.6 nm
Cascade50 17.7 nm
B
Spacer length
– 26 32 38 44 50 (nt)
TS cleavage (IR700 fluorescence)
NTS cleavage (IR800 fluorescence)
Uncut
Cut
C
Cascade32
Cascade38
Cascade44
Cascade50
No Cascade
HindIII
NTS
TS
Reads (x10³)
Reads (x10²)
D
PAM-distal
Cleavage
DNA
HNH
Cas5
Cas7.6
Cas8
PAM-proximal
Cas6
Cas7.1
Cas7.2
Cas7.3
Cas7.5
Cas7.4
5′
PAM
crRNA32
HNH
Nicking
Cas7.9
Cas7.8
Cas7.7
Cas7.3
Cas7.2
Cas7.1
crRNA50

Figure S9

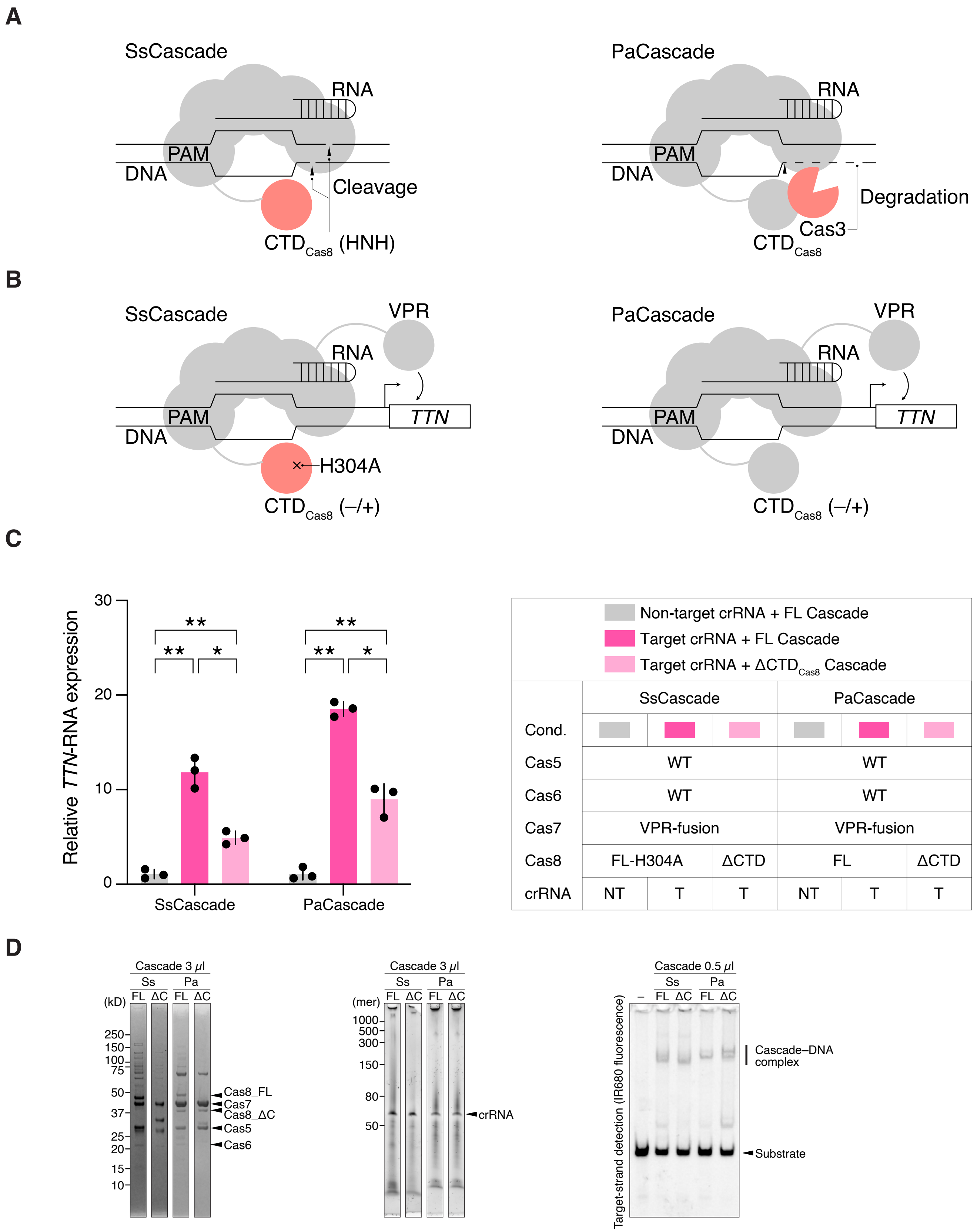
A
SsCascade
RNA
PAM
DNA
Cleavage
CTDCas8 (HNH)
PaCascade
RNA
PAM
DNA
Degradation
Cas3
CTDCas8
B
SsCascade
VPR
RNA
PAM
DNA
TTN
H304A
CTDCas8 (–/+)
PaCascade
VPR
RNA
PAM
DNA
TTN
CTDCas8 (–/+)
C
Relative TTN-RNA expression
30
20
10
0
**
**
*
SsCascade
PaCascade
Non-target crRNA + FL Cascade
Target crRNA + FL Cascade
Target crRNA + ΔCTDCas8 Cascade
Cond.
Cas5 WT WT
Cas6 WT WT
Cas7 VPR-fusion VPR-fusion
Cas8 FL-H304A ΔCTD FL ΔCTD
crRNA NT T T NT T T
D
Cascade 3 μl
Ss Pa
FL ΔC FL ΔC
(kD)
250 150 100 75 50 37 25 20 15 10
Cas8_FL
Cas7
Cas8_ΔC
Cas5
Cas6
(mer)
1000 500 300 150 80 50
crRNA
Cascade 0.5 μl
–
Target-strand detection (IR680 fluorescence)
Cascade–DNA complex
Substrate

Figure S10

A

	Cascade	EndoVII	I-PpoI
Targeting	RNA-guided	NA (Junction-specific)	Protein-guided
Function	Double strand break	Nick + Nick	
Formation	Monomer	Dimer	

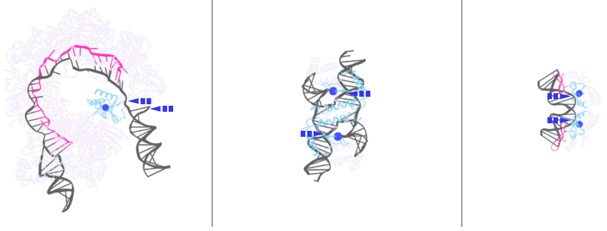

B

Cascade — Cas9

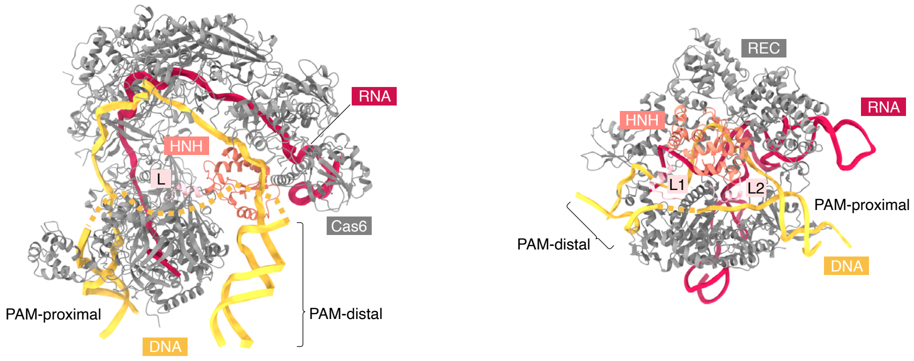

Figure S11
